# Novel Sulfonate Derivatives Functionalized with Triazole–Hydrazone Moieties: Synthesis, Characterization, DFT, Targeting Brain Tumors via DNA Damage, Cytotoxicity, Migration Suppression, Antimicrobial Activity, and In Silico Study

**DOI:** 10.3390/molecules31132281

**Published:** 2026-06-30

**Authors:** Yasemin Ünver, Meryem Evecen, Fatih Çelik, Ali Aydın, Halil İbrahim Güler, Kadriye İnan Bektaş, Tuğba Usta

**Affiliations:** 1 Department of Chemistry, Faculty of Sciences, Karadeniz Technical University, 61080 Trabzon, Turkey; fatih.celik502@gmail.com (F.Ç.); tugbausta93@gmail.com (T.U.); 2 Department of Electric and Electronic Engineering, Faculty of Engineering, Amasya University, 05100 Amasya, Turkey; meryem.evecen@amasya.edu.tr; 3 Department of Basic Medical Science, Faculty of Medicine, Bozok University, 66100 Yozgat, Turkey; ali.aydin@yobu.edu.tr; 4 Department of Molecular Biology and Genetics, Faculty of Science, Karadeniz Technical University, 61080 Trabzon, Turkey; hiboguler@gmail.com (H.İ.G.); kadriyensis@gmail.com (K.İ.B.); 5 Department of Chemistry, Graduate School of Natural and Applied Science, Karadeniz Technical University, 61080 Trabzon, Turkey

**Keywords:** triazole, sulfonate, hydrazone, IR and NMR spectroscopy, DFT, antitumor-antimicrobial activities, molecular docking

## Abstract

In this study, a new series of (E)-4-((2-(2-(4-amino-3-methyl-5-oxo-4,5-dihydro-1H-1,2,4-triazol-1-yl)acetyl)hydrazono)methyl)phenyl 4-halogenobenzenesulfonates (**3a**–**3d**), where **3a** = F, **3b** = Cl, **3c** = Br, and **3d** = I, were successfully synthesized via a straightforward synthetic route. The structures of the obtained compounds were fully characterized and confirmed by spectroscopic techniques, including FT-IR, ^1^H NMR, and ^13^C NMR, as well as LC-MS/MS analysis. 1,2,4-triazole-based hydrazone derivatives (**3a**–**3d**) were investigated using IR and NMR spectroscopy and DFT calculations. Intermolecular interactions, HOMO-LUMO, dipole moment, polarization, first-order hyperpolarizability, and molecular electrostatic potential studies on the molecules were examined. The HOMO and LUMO energy gap study supports the charge transfer probability in the molecules. These were conducted to investigate the reactivity and stability of heterocyclic molecules in bioactivity analysis. Electron density mapping within the molecular electrostatic potential plot and electrostatic potential representation within the iso-surface plot evaluated the concept of charge distribution in the molecule as nucleophilic reactions and electrophilic regions. The predicted nonlinear optical (NLO) properties of the molecules are much greater than those of urea. The results obtained from these investigations collectively provide evidence that the molecules possess nonlinear optical applications. Novel triazole–hydrazone-functionalized aryl sulfonate derivatives (**3a**–**3d**) were evaluated for their anticancer potential against a panel of brain and non-brain cancer cell lines. Compound **3b** exhibited the most favorable overall biological profile, displaying potent activity against SH-SY5Y neuroblastoma (GI = 7.59 μM) and U87MG glioblastoma cells (GI = 13.85 μM), together with the lowest toxicity toward normal FL fibroblasts (GI = 62.02 μM). Compounds **3c** and **3d** demonstrated remarkable potency against IDHmut-U87 glioma cells (GI = 3.87 and 3.27 μM, respectively), although their selectivity toward cancer cells was limited. DNA degradation studies revealed substantial fragmentation, particularly in C6 and SH-SY5Y cells, while migration assays indicated reduced cellular motility. Molecular docking studies identified compound **3b** as the strongest PI3Kα binder, supporting a possible. In addition, the antimicrobial activities of compounds **3a**–**3d** were evaluated against selected Gram-positive and Gram-negative bacteria as well as Candida species using the broth microdilution method. The compounds exhibited measurable antimicrobial effects with MIC values ranging from 156 to 625 µg/mL, showing moderate growth inhibition against the tested microorganisms. Although the observed activity was lower than that of the reference antimicrobial agents, the results indicate that these triazole–hydrazone derivatives possess a detectable level of antimicrobial activity and provide a basis for further structural optimization. Collectively, the results suggest that compound **3b** represents the most promising lead structure due to its balanced combination of potency, selectivity, and predicted target engagement. Molecular docking was performed to evaluate the binding potential of newly synthesized triazole derivatives (**3a**–**3d**) against PI3Kα. The docking protocol was validated by re-docking alpelisib, yielding an RMSD of 0.64 Å. Among the tested compounds, **3b** showed the most favorable binding energy (−9.94 kcal/mol) and estimated Ki value (52.13 nM), consistent with its superior in vitro activity. Its interactions with key PI3Kα residues, including Val851, Ser854, Met922, and Asp933, support a stable binding mode within the ATP-binding pocket. In silico ADME and toxicity analyses suggested acceptable drug-likeness characteristics, absence of major hepatotoxic, mutagenic, and carcinogenic liabilities, and moderate predicted acute toxicity profiles. These findings suggest that **3b** is the most promising derivative for further validation.

## 1. Introduction

Heterocyclic organic compounds rich in functional groups continue to attract considerable research interest due to their versatile physicochemical and biological properties. In this context, the incorporation of different functional groups within a single molecular framework enables the design of compounds with novel and enhanced properties [1,2,3]. In particular, molecules containing triazole, hydrazone, and halogenated sulfonate groups exhibit remarkable potential applications in interdisciplinary fields such as pharmaceutical chemistry, dye chemistry, agrochemicals, and materials science [4,5]. Triazole derivatives have gained significant importance in both biological and industrial applications owing to their aromatic structures and high thermal stability [6,7,8,9,10]. The hydrazone group, due to the presence of the C=N bond and its conjugated structure, provides electronic delocalization and structural flexibility to molecular systems [11]. Hydrazone derivatives display a broad spectrum of biological activities, particularly antimicrobial, antitubercular, and antioxidant properties. In addition, hydrazone compounds are widely utilized in molecular recognition, sensor systems, and coordination chemistry, owing to their tautomeric behavior and hydrogen-bonding capability [11,12,13,14,15,16,17,18,19]. Sulfonate groups are among the key functional groups that enhance the water solubility of compounds due to their high polarity and ionic character [20]. In particular, sulfonate structures substituted with halogen atoms allow controlled modulation of chemical reactivity and stability through strong electron-withdrawing effects [21]. Halogenated sulfonate-containing compounds are widely employed in the design of reactive dyes in dye and textile chemistry, and they have also attracted attention in pharmaceutical applications due to their surfactant properties and interactions with biological systems [22,23,24]. The presence of halogen atoms plays a decisive role in lipophilicity and bioavailability [25,26,27,28,29]. In this context, a new series of (E)-4-((2-(2-(4-amino-3-methyl-5-oxo-4,5-dihydro-1H-1,2,4-triazol-1-yl)acetyl)hydrazono)methyl)phenyl 4-halogenobenzenesulfonates (**3a**–**3d**), where (**3a** = F, **3b** = Cl, **3c** = Br, and **3d** = I), were synthesized and structurally characterized by FT-IR, ^1^H NMR, ^13^C NMR, and LC-MS/MS analyses. Furthermore, density functional theory (DFT) calculations were performed to evaluate the electronic structures, HOMO–LUMO energies, molecular electrostatic potential distributions, and nonlinear optical (NLO) properties of the synthesized compounds. In addition, their anticancer potential was investigated through comprehensive in vitro biological studies, including antiproliferative activity, membrane toxicity, DNA-binding and DNA degradation analyses, migration assays, and morphological evaluations against various cancer cell lines. The antimicrobial activities of compounds **3a**–**3d** were evaluated against selected Gram-positive and Gram-negative bacteria as well as *Candida* species using the broth microdilution method. To gain preliminary insights into potential molecular interactions associated with the observed antiproliferative activity, molecular docking studies were performed against PI3Kα, a cancer-related molecular target known to regulate tumor growth, survival, and migration. The docking analysis was intended as an exploratory computational approach to investigate possible ligand–target interactions rather than to establish PI3Kα inhibition as the definitive mechanism of action.

## 2. Results

### 2.1. Chemistry

The synthesized (E)-configured 1,2,4-triazole-based hydrazone derivatives (**3a**–**3d**) (**3a** = F, **3b** = Cl, **3c** = Br, and **3d** = I) were obtained in good to excellent yields (75–90%) via condensation of 4-formylphenyl 4-halogenbenzenesulfonates with 2-(4-amino-3-methyl-5-oxo-4,5-dihydro-1H-1,2,4-triazol-1-yl)acetohydrazide under reflux in ethanol. The smooth progress of the reaction and satisfactory yields indicate that the aldehyde and hydrazide functionalities readily undergo Schiff base formation under the applied conditions. Among the derivatives, the iodo-substituted compound (**3d**) afforded the highest yield, whereas the bromo analogue (**3c**) gave the lowest yield, which may be attributed to steric and electronic differences in the halogen substituents influencing the electrophilicity of the carbonyl carbon and overall reaction efficiency.

The IR spectra of compounds **3a**–**3d** confirm the successful formation of the target hydrazone structures. The characteristic absorption bands observed at 3320–3279 cm^−1^ correspond to the asymmetric and symmetric stretching vibrations of the NH_2_ group, while the NH proton stretching appeared in the 3095–3109 cm^−1^ region. The presence of two distinct carbonyl stretching bands at 1720–1692 cm^−1^ is consistent with the triazole C=O and acyl hydrazide C=O groups. The azomethine (C=N) stretching vibration observed at 1608–1626 cm^−1^ clearly indicates the formation of the hydrazone linkage. Additionally, aromatic C=C stretching bands detected in the range of 1566–1592 cm^−1^ further support the presence of substituted phenyl rings. The consistency of these absorption bands across all derivatives confirms the structural similarity of the series.

The ^1^H NMR spectra provide further structural evidence. In all compounds, the methyl group attached to the triazole ring appeared as a singlet at 2.12 ppm (3H), while the methylene (N–CH_2_) protons were observed as singlets at 4.39–4.80 ppm (2H), confirming the presence of the acetyl linker. The NH_2_ protons resonated as a singlet at 5.31–5.32 ppm (2H), and the hydrazide NH proton appeared as a downfield singlet at 11.72–11.73 ppm, suggesting hydrogen bonding effects in DMSO-d_6_. The azomethine proton (N=CH) signal observed in the aromatic region supports the formation of the Schiff base structure. The aromatic protons of both phenyl rings appeared in the expected region between 7.10 and 8.18 ppm, with slight variations depending on the nature of the halogen substituent, reflecting the electronic influence of F, Cl, Br, and I atoms on the aromatic system. The ^13^C NMR spectra are also in good agreement with the proposed structures. The methyl carbon of the triazole ring was observed at 11 ppm, and the methylene carbon at δ ≈ 46 ppm. The azomethine carbon resonated at 142–145 ppm, while the triazole carbonyl carbon appeared at 150 ppm. The acyl carbonyl carbon signal was detected at 168–169 ppm. The aromatic carbon signals showed characteristic shifts influenced by the halogen substituents, particularly in the para-halogenated phenyl rings, confirming the successful incorporation of the respective substituents. Overall, the combined IR and NMR spectral data unambiguously confirm the successful synthesis of the desired (E)-hydrazone-linked 1,2,4-triazole derivatives bearing different halogen-substituted benzenesulfonate moieties. The spectral similarities across the series, along with predictable substituent-induced variations, support the proposed molecular structures.

### 2.2. Optimized Structure

**Figure 1:**
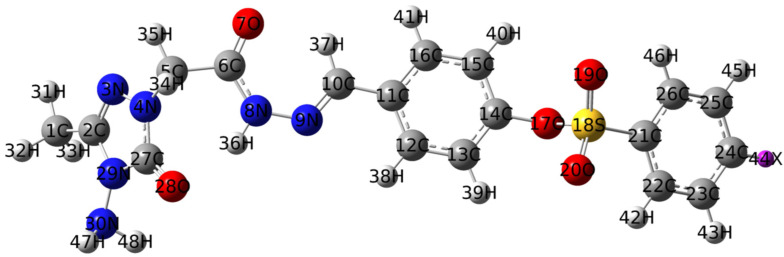
Optimized structure of compounds, C_18_H_17_N_6_O_5_SX (X: F, Cl, Br, and I).

Structural information is useful when investigating the coordination properties of Schiff bases functioning as ligands. We report here the synthesis and molecular structure of the title Schiff base compounds, chemical formula C_18_H_17_N_6_O_5_SX (X: F, Cl, Br, and I), and the atom numbering for them is shown in Figure 1. First, the initial molecular geometry of molecules was obtained using the Gauss-View 05 software program in quantum chemical calculations. The optimized energy value was found as −41,097 eV for molecule **3a**, which was smaller than the others (E_3b_ = −38,786 eV, E_3c_ = −38,738 eV, E_3d_ = −38,689 eV). We made geometry optimizations for molecules using B3LYP and comparative optimized structural parameters tabulated in Table S1. The focus on comparing the structural parameters of optimized molecular structures is to observe the inductive effect of halogenated sulfonate functional groups. By attaching different halogens to the phenylene ring in the compounds, differences were observed only in the C24–F44, C24–Cl44, C24–Br44, and C24–I44 bonds as 1.40 (exp. 1.352 [30]), 1.81 (exp. 1.737 [31]), 1.96 (exp. 1.900 [32,33]), and 2.14 Å (exp. 2.097 [34], respectively. This difference increased in bond angles, and it was observed that the greatest inductive effect occurred in dihedral angles. C–C–C angles remain close to 120° in most cases, supporting trigonal planar (sp^2^) hybridization. In all compounds, the O–S–O angle was measured at approximately 120°. The C5-N4-C27 bond angle is approximately 126°, indicating delocalized electron density around the nitrogen centers. The C23-C24-X44 angles vary around 118°–120° due to the electronegativity and steric hindrance of the halogens. The widest bond angle in the system is approximately 129.3° (C6-N8-N9), while the smallest bond angle is approximately 96° (O17-S18-C21). Dihedral angles reveal the three-dimensional conformation of the molecule. The highest dihedral angle is approximately 179.9°, while the lowest dihedral angle is approximately −179.9°, both indicating nearly planar conformations. The dihedral angles show a mixture of planar and twisted conformations, indicating flexibility in molecular geometry. These findings are important for understanding the reactivity, stability, and potential applications of the molecule in chemical or biological systems. The results obtained are consistent with similar molecules [35,36,37,38].

### 2.3. Spectroscopic Properties

#### 2.3.1. Vibrational Spectra

The IR spectra of compounds **3a**–**3d** (**3a** = F, **3b** = Cl, **3c** = Br, and **3d** = I) confirmed the successful formation of the target hydrazone structures. Characteristic NH_2_ and NH stretching vibrations were observed at 3320–3279 and 3095–3109 cm^−1^, respectively, in reasonable agreement with the calculated values of 3466–3592 and 3380–3387 cm^−1^. Aromatic C–H stretching vibrations appeared at 3073–3109 cm^−1^. Two carbonyl stretching bands at 1720–1692 cm^−1^ were assigned to triazole and acyl hydrazide C=O groups, consistent with theoretical values of 1646 and 1640 cm^−1^. The azomethine (C=N) stretching vibration observed at 1608–1626 cm^−1^ confirmed hydrazone formation and appeared as a characteristic band at 1595 cm^−1^ for all compounds, indicating strong conjugation within the molecular framework [39,40]. Aromatic C=C stretching bands were detected at 1566–1592 cm^−1^. The asymmetric SO_2_ stretching vibrations appeared at 1290–1311 and 825 cm^−1^, in agreement with literature reports [38,41,42]. The experimental and theoretical vibrational data are summarized in Table 1, and the overall consistency of the bands confirms the structural similarity of the series.

#### 2.3.2. NMR Studies

The ^1^H and ^13^C NMR chemical shift values of (**3a**–**3d**) (**3a** = F, **3b** = Cl, **3c** = Br, and **3d** = I) molecules were computed at the same level using the GIAO approach in DMSO solvent media, based upon the compound’s optimized molecular geometries. The experimentally obtained and computed chemical shifts of the mentioned molecule are presented in Table 2 and Table 3. The ^1^H NMR spectra provide further structural evidence. In all compounds, the methyl group attached to the triazole ring appeared as a singlet at 2.12 ppm (3H), while the methylene (N–CH_2_) protons were observed as a singlet at 4.39–4.80 ppm (2H), confirming the presence of the acetyl linker. These values were theoretically recorded in the ranges of 2.27–2.48 ppm (3H) and 4.29–4.79 ppm (2H), respectively. In accordance with the theoretical values, the NH_2_ protons resonated as a singlet at 5.31–5.32 ppm (2H), and the hydrazide NH proton appeared as a downfield singlet at 11.72–11.73 ppm; this suggests hydrogen bonding effects in DMSO-d_6_. The azomethine proton (N=CH) signal observed in the aromatic region supports the formation of the Schiff base structure. The aromatic protons of both phenyl rings appeared in the expected region between 7.10 and 8.18 ppm experimentally and 7.88–8.82 ppm theoretically, showing slight variations depending on the nature of the halogen substituent, reflecting the electronic effect of F, Cl, Br, and I atoms on the aromatic system (Table 2).

The ^13^C NMR spectra are also in good agreement with the proposed structures (Table 3). The methyl carbon of the triazole ring was observed at 11 ppm, while the methylene carbon was observed at δ ≈ 46 ppm. The azomethine carbon resonated at 142–145 ppm, while the triazole carbonyl carbon appeared at 150 ppm. Similarly, the ^13^C-NMR chemical shift values for these carbon atoms were recorded at 162 ppm and 167 ppm, respectively. The acyl carbonyl carbon signal was detected at 168–169 ppm. The aromatic carbon signals exhibited characteristic shifts, particularly in the para-halogenated phenyl rings due to the effect of halogen substituents, confirming the successful incorporation of the respective substituents.

Overall, the combined IR and NMR spectral data unambiguously confirm the successful synthesis of the desired (E)-hydrazone-linked 1,2,4-triazole derivatives bearing different halogen-substituted benzenesulfonate moieties. The spectral similarities across the series, along with predictable substituent-induced variations, support the proposed molecular structures.

### 2.4. Electronic Properties

#### 2.4.1. Molecular Orbital Calculations

**Figure 2:**
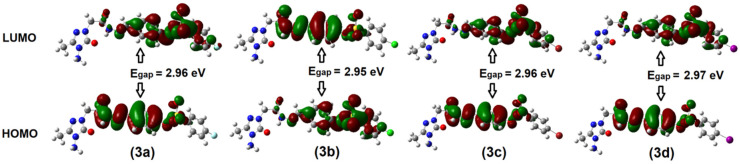
Frontier molecular orbitals for compounds.

Frontier Molecular Orbital (FMO) analysis is an important tool for understanding the electronic properties of molecules, particularly their chemical reactivity and stability [43]. The FMO surface map is shown in Figure 2. In Figure 2, green represents the positive phase, while red represents the negative phase. Molecules with a larger energy gap (ΔE) exhibit higher chemical stability and lower reactivity. HOMO and LUMO energies, together with the energy gap (ΔE), provide information about electronic transitions, ionization potential, electron affinity, and other global reactivity descriptors [44]. The global reactivity descriptors are presented comparatively in Table 4.

Molecule (**3a**), which has the lowest HOMO energy, also has a lower tendency to donate electrons. Similarly, molecule (**3c**) with lower LUMO energy has a higher electron-accepting ability. Therefore, it can be stated that molecule (**3a**) requires more energy for ionization and exhibits stronger resistance to oxidation [45]. Molecule (**3d**), which has a high hardness (η) value, is chemically more stable while having the lowest electronegativity value. The chemical potential (μ) value, which indicates a molecule’s tendency to gain or lose electrons, is again highest in molecule **3a**. Furthermore, molecule **3b**, which has the highest electrophilicity and nucleophilicity indices, has greater electron-accepting and electron-donating capacity, respectively. These findings provide valuable information about the electronic properties and reactivity of the investigated compounds.

#### 2.4.2. MEP Analysis

**Figure 3:**
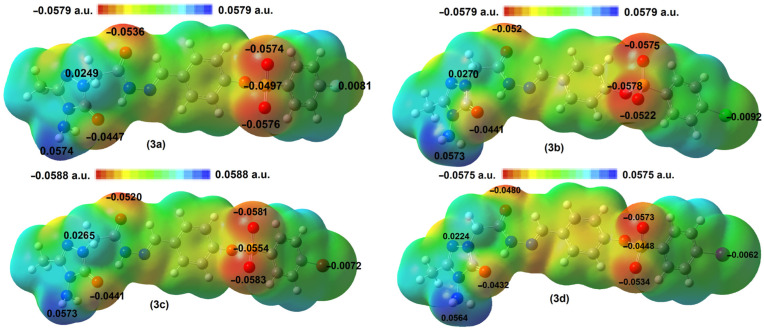
MEP surfaces of (**3a**–**3d**) compounds.

Molecular Electrostatic Potential (MEP) analysis is a tool used to understand the charge distribution, electrophilic and nucleophilic regions, and overall reactivity of a molecule [46]. In the MEP map, different colors represent positive and negative electrostatic potential regions. The MEP color surface map is shown in Figure 3. Blue regions correspond to areas with electron deficiency, while red regions represent electron-rich areas. In this study, the nitro and oxygen groups exhibit greater reactivity. Particularly, the presence of the chlorine (-Cl) substituent slightly withdraws electron density, affecting the overall charge distribution and leading to a more stable electrostatic potential. These observations are important in understanding the electronic behavior of molecules and their potential applications in chemical and biological interactions.

#### 2.4.3. NLO Properties

Molecules with large hyperpolarizability can be used as beneficial optical devices. As shown in Table 5, the high hyperpolarizability value corresponds to a large HOMO-LUMO energy gap (ΔE). This indicates that there is a direct relationship between ΔE and hyperpolarizability (Figure 2). The total dipole moment (μ), average polarizability (α), anisotropy of the polarizability (Δα), and total first-order hyperpolarizability (β) values were calculated for molecules **3a**–**3d** and are presented in Table 5. Urea is commonly used as a threshold value for comparison purposes. The β value is estimated as 115.847 × 10^−29^, 112.527 × 10^−30^, 116.723 × 10^−30^, and 118.680 × 10^−30^ cm^5^/esu for **3a**–**3d**, respectively, which is larger than the value for urea (α and β value were 3.88 D, 5.04 Å^3^ and 0.782 × 10^−30^ cm^5^/esu) [37]. Therefore, the studied compounds may be attractive materials for nonlinear optical applications.

### 2.5. MTT Proliferation Assay

**Figure 4:**
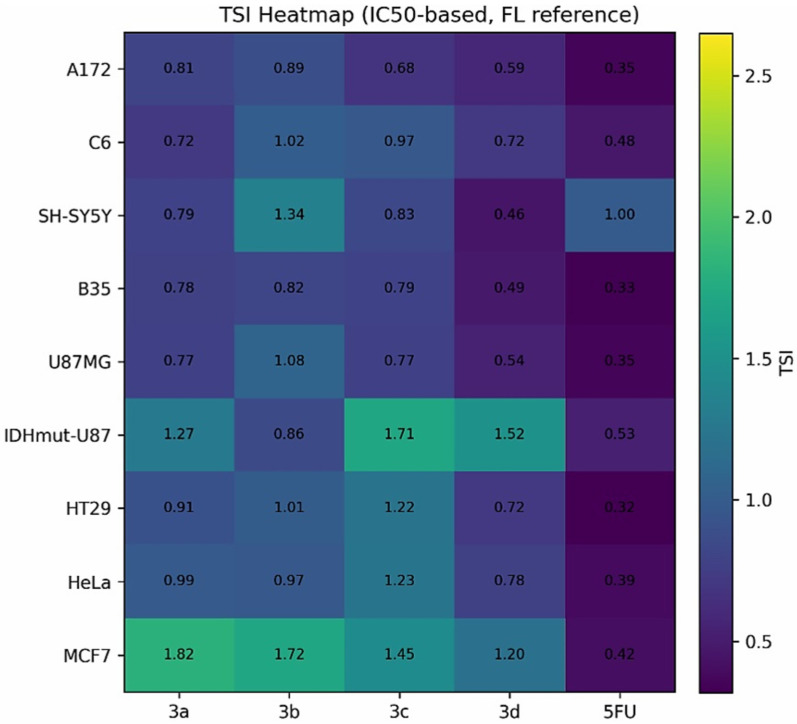
IC_50_-based tumor selectivity index (TSI) heatmap of compounds relative to FL cells. Higher TSI values indicate greater cancer cell selectivity.

The antiproliferative effects of compounds **3a**–**3d** were evaluated against a panel of brain and non-brain cancer cell lines using the MTT assay and compared with 5-fluorouracil (5-FU). Distinct differences in cellular sensitivity were observed among the tested compounds and cell types. Brain tumor models included A172 and U87MG human glioblastoma cells, IDHmut-U87 glioma cells, C6 rat glioma cells, SH-SY5Y human neuroblastoma cells, and B35 rat neuroblastoma cells. To assess the broader anticancer potential of the synthesized compounds, HT29 colon adenocarcinoma, HeLa cervical carcinoma, and MCF7 breast adenocarcinoma cells were also included. In addition, FL fibroblasts were used as a non-transformed cell model to evaluate selectivity for cancer cells and to estimate potential toxicity to normal tissues. The inclusion of both malignant and non-malignant cell lines allowed a comparative assessment of anticancer efficacy and tumor selectivity, with particular emphasis on brain tumor-related malignancies. GI_50_ and IC values were determined to provide complementary information regarding the biological effects of the tested compounds. GI_50_ represents the concentration required to inhibit net cell growth by 50%, reflecting antiproliferative (cytostatic) activity, whereas IC_50_ corresponds to the concentration required to reduce cell viability by 50% relative to untreated controls, reflecting overall cytotoxic activity. Since these parameters describe different aspects of cellular response, both were included in the present study. Among the brain tumor models, compound **3b** exhibited the most favorable activity profile, showing potent growth inhibition against SH-SY5Y neuroblastoma cells (GI_50_ = 7.59 μM) and U87MG glioblastoma cells (GI_50_ = 13.85 μM) (Table 6). In contrast, compounds **3c** and **3d** demonstrated superior activity against IDHmut-U87 glioma cells, with GI_50_ values of 3.87 and 3.27 μM, respectively, exceeding the activity of 5-FU (GI_50_ = 14.15 μM) (Table 6). Compound **3a** showed notable potency against MCF7 breast cancer cells (GI_50_ = 5.60 μM), while compounds **3c** and **3d** also exhibited strong activity against HT29 colon cancer cells (GI_50_ = 3.71 and 22.81 μM, respectively) (Table 7). Evaluation of FL fibroblasts revealed marked differences in selectivity among the synthesized derivatives. Compound **3b** displayed the lowest toxicity toward normal cells (GI_50_ = 62.02 μM), whereas compounds **3c** and **3d** showed considerably lower FL GI_50_ values (6.13 and 4.96 μM, respectively) (Table 7). Overall, the molar-normalized data identified compound **3b** as the most balanced derivative, combining potent antiproliferative activity with improved selectivity toward cancer cells, while compounds **3c** and **3d** emerged as the most potent but less selective analogues. Consistent with the biological findings, molecular docking studies identified compound **3b** as the derivative with the highest predicted affinity toward PI3Kα. Tumor selectivity was evaluated using the Tumor Selectivity Index (TSI), calculated according to the following equation: TSI = IC_50_ (FL normal fibroblasts)/IC_50_ (cancer cell line). A TSI value greater than 1 indicates preferential activity toward cancer cells, whereas higher TSI values reflect greater tumor selectivity and a potentially improved therapeutic window. Tumor selectivity index (TSI) analysis demonstrated the highest selectivity for compound **3c** in IDHmut-U87 cells (TSI = 1.71), followed by compound **3d** in the same cell line (TSI = 1.52). Compound **3a** exhibited favorable selectivity toward MCF7 breast cancer cells (TSI = 1.82) (Figure 4).

### 2.6. LDH Cytotoxicity Assay

The cytotoxic effects of compounds **3a**–**3d**, where **3a** = F, **3b** = Cl, **3c** = Br, and **3d** = I, were evaluated by LDH release assay at their respective IC concentrations across multiple cancer cell lines and FL normal fibroblast cells. LDH release analysis revealed relatively low membrane-disruptive effects for all synthesized compounds. Cytotoxicity values ranged between 3.08% and 12.44%, substantially lower than those observed for 5-FU (Table 8). The highest LDH release was detected for compound **3d** in MCF7 cells (12.44%), followed by compound **3a** (12.31%) and compound **3c** (11.99%) in the same cell line (Table 8). In contrast, compounds generally produced low LDH release in brain cancer models. Compound **3d** exhibited particularly low cytotoxicity in U87MG cells (3.28%), whereas compound **3b** showed the lowest value in HT29 cells (3.08%) (Table 8). These findings indicate that the observed growth inhibitory effects are unlikely to result primarily from acute membrane damage.

### 2.7. DNA Degradation Assay

**Figure 5:**
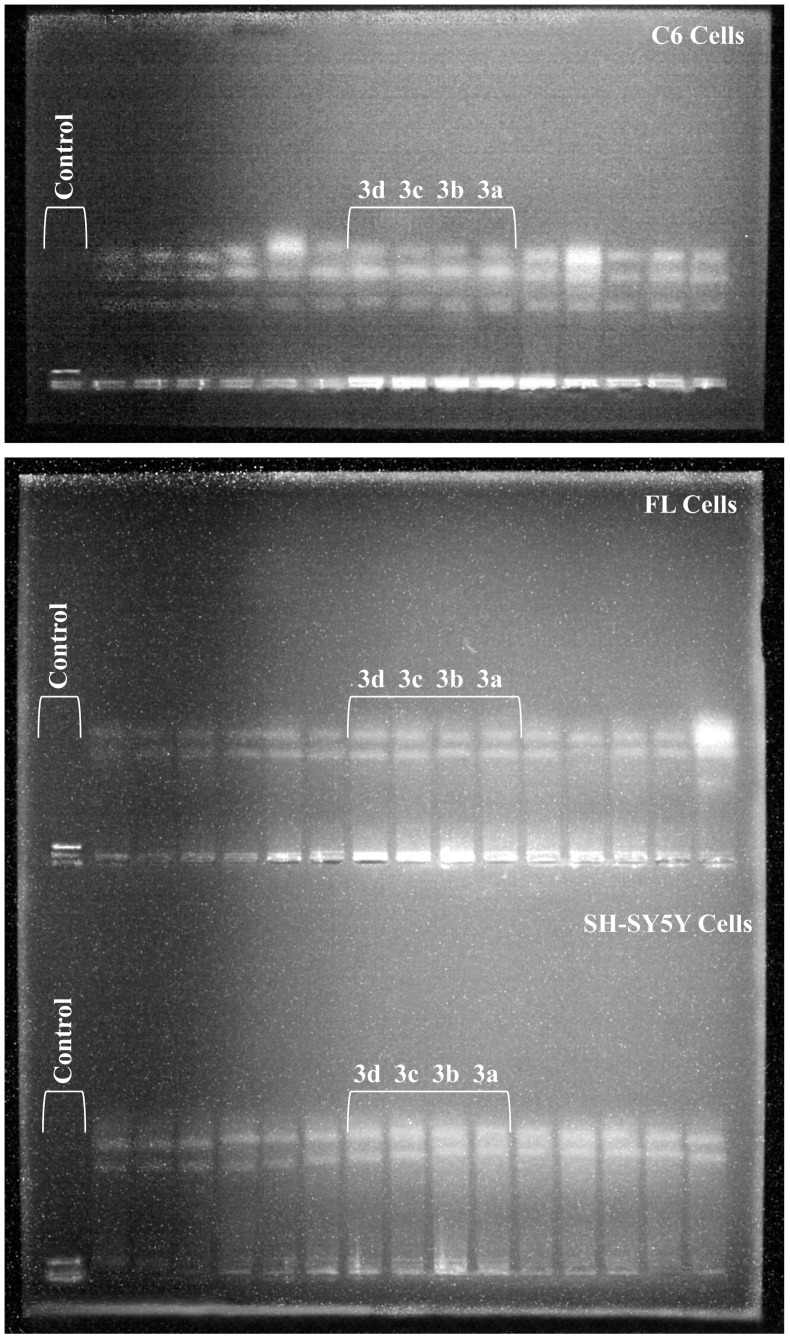
Test results demonstrating the DNA degradation effects of the **3**(**a**–**d**) on C6, FL, and SH-SY5Y cell lines.

DNA fragmentation analysis revealed that compounds **3a**–**3d**, where **3a** = F, **3b** = Cl, **3c** = Br, and **3d** = I, induced marked DNA degradation in treated cells, with the extent of degradation varying among cell lines. The most prominent DNA damage pattern was observed in C6 cells, where all tested compounds produced extensive DNA fragmentation/degradation. This finding indicates that C6 cells were highly sensitive to the DNA-damaging or apoptosis-associated effects of these compounds (Figure 5).

A strong DNA degradation profile was also detected in SH-SY5Y cells, although the intensity of DNA fragmentation appeared slightly lower than that observed in C6 cells. This suggests that SH-SY5Y cells were also responsive to treatment, but with a comparatively lower degree of DNA breakdown (Figure 5). In FL normal fibroblast cells, DNA degradation was also observed after treatment with the tested compounds. However, the degree of DNA damage appeared relatively lower than that detected in C6 and SH-SY5Y cancer cells. These results suggest that the compounds may induce DNA fragmentation preferentially in tumor cells, particularly in C6 and SH-SY5Y cells, while producing a comparatively weaker effect in FL cells (Figure 5).

Overall, DNA gel electrophoresis demonstrated that compounds **3a**–**3d** caused substantial DNA degradation, especially in C6 cells, followed by SH-SY5Y cells, whereas FL cells showed a relatively lower level of DNA fragmentation.

### 2.8. Influence of Molecules on Cell Migration and ImageR Evaluation

**Figure 6:**
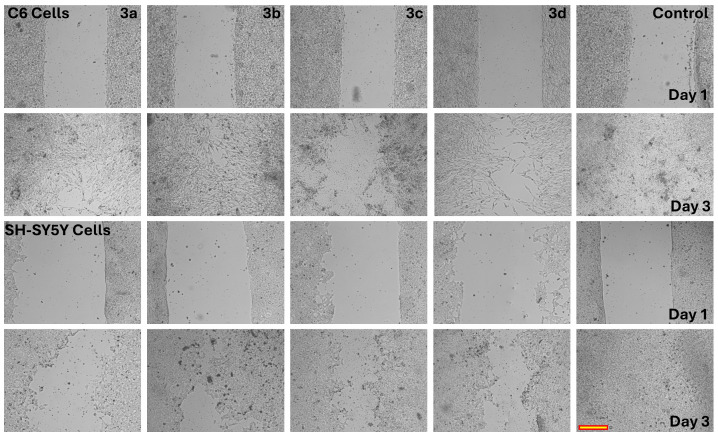
Effects of the **3**(**a**–**d**) on cell migration in the C6 and SH-SY5Y cancer cell lines. Bar is 100 µm.

Cell migration was assessed using wound-healing analysis by calculating the reduction in wound area between Day 1 and Day 3. The gap-filling ratio represented the percentage decrease in the wound area over the experimental period. In control groups, complete closure of the wound area was observed by Day 3, resulting in a Day 3 gap value of 0%, indicating unrestricted migratory capacity (Figure 6).

In C6 cells, compound **3c** produced the strongest anti-migratory effect, showing the lowest gap-filling ratio (24.71%), followed by compound **3d** (29.86%) (Table 9). Compounds **3a** and **3b** displayed moderate inhibition with gap-filling ratios of 32.88% and 35.13%, respectively (Table 9). In SH-SY5Y cells, compound **3a** showed the strongest inhibition of migration (20.24%), followed by compound **3c** (32.54%) and compound **3d** (34.14%) (Table 9). Compound **3b** displayed comparatively weaker suppression (39.84%) (Table 9). These findings demonstrate that all compounds interfere with cellular migration, although their efficacy appears dependent on cell type (Table 9 and Figure 6).

### 2.9. DNA Binding Assay

**Figure 7:**
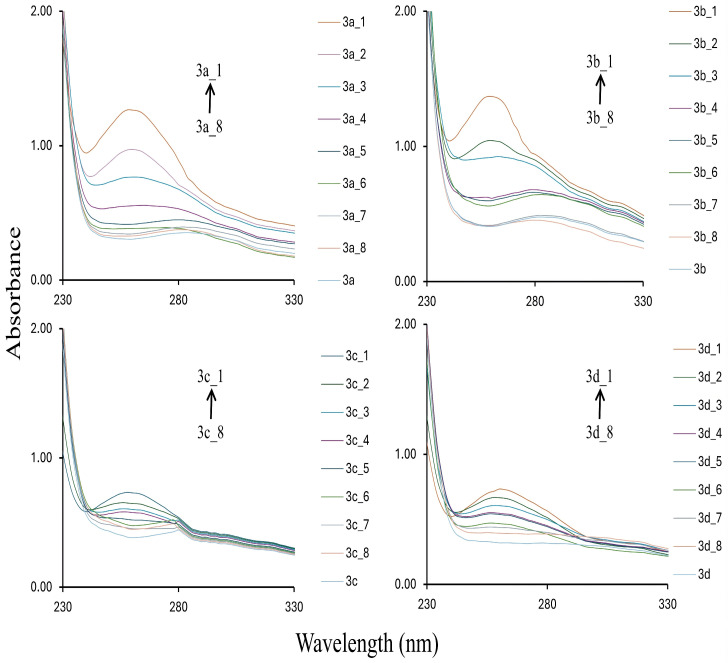
UV–Vis absorption spectrum of **3a**–**d** in the absence and presence of DNA [3.125 µM (8), 6.25 µM (7), 12.5 µM (6) 25 µM (5), 50 µM (4), 100 µM (3), 200 µM (2), and 400 μM (1)] of the UV–Vis absorption spectrum.

**Figure 8:**
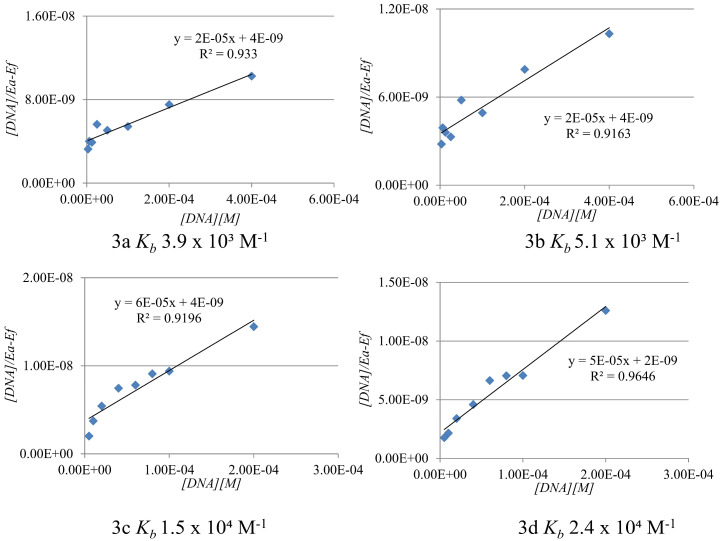
Plot chart indicating binding constant [DNA] and [DNA]/εa − εf.

The DNA-binding properties of compounds **3a**–**3d** were investigated using UV–Vis absorption spectroscopy and evaluated by Wolfe–Shimer analysis. Progressive addition of CT-DNA induced notable alterations in the absorption spectra of all tested compounds, indicating interactions between the molecules and DNA (Figure 7). Upon increasing DNA concentration, compounds generally exhibited concentration-dependent spectral changes characterized by reductions in absorption intensity together with moderate spectral perturbations in the 250–300 nm region. The gradual changes observed between the first (1) and last (8) DNA concentrations indicate formation of compound–DNA complexes (Figure 7). The calculated binding constants (Kb) varied among compounds, suggesting differences in binding affinity toward DNA: **3a**: 3.9 × 10^3^ M^−1^, **3b**: 5.1 × 10^3^ M^−1^, **3c**: 1.5 × 10 M^−1^, and **3d**: 2.4 × 10 M^−1^ (Figure 7). Among the tested molecules, compound **3d** exhibited the strongest DNA-binding affinity, followed by compound **3c**. The Wolfe–Shimer plots showed satisfactory linearity (R^2^ = 0.916–0.993), supporting the reliability of the calculated binding constants (Figure 8).

Upon increasing DNA concentration, all compounds exhibited a noticeable hyperchromic effect, characterized by a gradual increase in absorbance intensity, suggesting the formation of compound–DNA complexes and alterations in the DNA microenvironment. The hyperchromic effect became more pronounced at higher DNA concentrations, indicating increased interactions between the compounds and DNA molecules. The observed spectral changes suggest that the compounds can influence DNA structural organization.

### 2.10. Morphological Analysis

**Figure 9:**
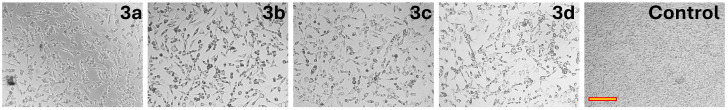
Morphological changes caused by **3a**–**d** at a concentration of 32 µg/mL in the U87MG cell line. Bar is 100 µm.

Morphological alterations induced by compounds **3a**–**3d**, where **3a** = F, **3b** = Cl, **3c** = Br, and **3d** = I, at 32 μg/mL were evaluated by phase-contrast microscopy and compared with untreated control cells. Control cells exhibited normal morphology characterized by a dense monolayer with high confluence, elongated spindle-like morphology, intact cell–cell contacts, and strong substrate attachment (Figure 9). In contrast, treatment with the tested compounds induced substantial morphological changes of varying intensity. Cells exposed to **3a** showed reduced confluence with the appearance of rounded and partially detached cells, accompanied by loss of normal cellular organization. Compound **3b** induced noticeable cell shrinkage and increased numbers of rounded cells while preserving some elongated cells (Figure 9). Treatment with **3c** and **3d** produced more pronounced morphological alterations characterized by increased cellular rounding, reduction in cell density, and disruption of normal cellular architecture. Detached cells and cells with irregular morphology were more evident, indicating progressive cellular damage (Figure 9). Overall, treated groups demonstrated decreased confluence, cellular shrinkage, loss of adhesion, increased rounding, and cellular detachment compared with untreated controls, with the strongest effects observed particularly in **3d** and **3c**.

The present study demonstrated that compounds **3a**–**3d** exert multifaceted anticancer effects through modulation of several cellular processes involved in tumor progression. MTT analysis revealed significant antiproliferative activities in different cancer cell lines, although responses varied depending on compound structure and cell type. While 5FU showed the strongest overall activity, several synthesized compounds exhibited considerable growth inhibition, suggesting that structural differences strongly influence biological activity.

Interestingly, LDH analysis showed relatively low cytotoxicity values compared with the marked reduction in cell viability observed in MTT results. This suggests that the biological effects of these compounds are not mainly associated with acute membrane damage or necrotic cell death but may instead involve intracellular regulatory pathways. Such discrepancies between MTT and LDH findings are frequently associated with cytostatic or programmed cell death mechanisms.

DNA-binding studies demonstrated that all compounds interacted with DNA with moderate affinity (Kb ≈ 10^3^–10^4^ M^−1^), with **3d** and **3c** exhibiting stronger interactions.

However, DNA-binding affinity did not completely correlate with antiproliferative activity, indicating that DNA interaction alone is insufficient to explain the observed biological effects.

DNA degradation analysis revealed substantial fragmentation, particularly in C6 cells, followed by SH-SY5Y cells, whereas FL cells showed comparatively lower degradation. These findings suggest a partially selective response toward cancer cells and support the involvement of apoptosis-associated mechanisms. Migration studies further showed that all compounds reduced wound closure in both C6 and SH-SY5Y cells, indicating potential anti-migratory activity. Since cell migration plays a critical role in metastasis, these findings suggest possible anti-invasive properties of the tested compounds.

Morphological observations supported these biochemical findings by demonstrating reduced confluence, cell rounding, shrinkage, loss of adhesion, and increased detached cells following treatment. Such changes are commonly associated with apoptosis-like responses and cellular stress.

Overall, the findings suggest that compounds **3a**–**3d** exert anticancer effects through multiple interconnected mechanisms involving proliferation suppression, DNA interaction, DNA degradation, migration inhibition, and induction of apoptosis-associated cellular changes. Further mechanistic studies involving apoptosis and signaling pathways would provide deeper insight into their therapeutic potential.

### 2.11. Antimicrobial Activity

The antimicrobial activities of compounds **3a**–**3d**, where **3a** = F, **3b** = Cl, **3c** = Br, and **3d** = I, were evaluated against selected Gram-positive and Gram-negative bacteria, as well as yeast strains, using the broth microdilution method. The minimum inhibitory concentration (MIC) values are presented in Table 10. Overall, the synthesized compounds exhibited measurable inhibitory effects against the tested microorganisms, with MIC values ranging from 156 to 625 µg/mL. Among the bacterial strains tested, all derivatives showed comparable inhibitory activity against *Bacillus cereus* and *Klebsiella pneumoniae*, with MIC values of 156 µg/mL. Moderate growth inhibition was also observed against *Bacillus subtilis*, *Escherichia coli*, *Pseudomonas aeruginosa*, and *Enterococcus faecalis*, with MIC values generally between 312 and 625 µg/mL. *Staphylococcus aureus* was found to be less susceptible to the tested compounds. The antifungal evaluation revealed that the compounds were also active against *Candida albicans* and *Candida tropicalis*, with MIC values ranging between 156 and 312 µg/mL. However, when compared with the reference antifungal agent fluconazole, the activity of the synthesized derivatives was lower. Nevertheless, the observed inhibitory effects indicate that these compounds retain a measurable level of antifungal activity. Comparison of the halogen-substituted derivatives showed only minor differences in antimicrobial performance, suggesting that substitution with F, Cl, Br, or I did not lead to substantial changes in activity under the tested conditions. The antimicrobial effects observed may be related to the combined contribution of the triazole, hydrazone, and halogenated sulfonate moieties within the molecular framework. Overall, the results indicate that the synthesized compounds display moderate antimicrobial activity against the tested microorganisms. Although their activity was lower than that of the reference antimicrobial agents, the findings provide preliminary insight into the biological behavior of this structural scaffold and may be useful for future structural modification studies aimed at improving antimicrobial efficacy.

### 2.12. Molecular Docking Analysis

Molecular docking analysis was performed to evaluate the binding potential of the newly synthesized triazole derivatives **3a**–**3d**, where **3a** = F, **3b** = Cl, **3c** = Br, and **3d** = I, against the ATP-binding pocket of PI3Kα. PI3Kα was selected as an exploratory molecular target because aberrant activation of the PI3K/Akt signaling pathway has been implicated in several cancer types represented by the cellular models used in this study, including glioblastoma, neuroblastoma, breast cancer, and colorectal cancer. Moreover, structurally related triazole- and hydrazone-containing compounds have previously been reported to exert anticancer effects through mechanisms associated with PI3K/Akt signaling. Therefore, docking studies were performed to investigate potential ligand–target interactions and provide preliminary mechanistic insights into the observed antiproliferative activity. Prior to ligand docking, the docking protocol was validated by re-docking the co-crystallized inhibitor, alpelisib, into the active site of PI3Kα. The re-docked pose showed an RMSD value of 0.64 Å relative to the crystallographic pose, confirming that the selected grid parameters and docking protocol were reliable for reproducing the experimentally observed binding orientation. Since RMSD values below 2.0 Å are generally accepted as an indicator of successful docking validation, this result supports the robustness of the docking workflow used in the present study [47,48].

The docking results showed that all synthesized derivatives were favorably accommodated within the PI3Kα catalytic pocket, with predicted binding energies ranging from −9.10 to −9.94 kcal/mol and estimated K_i_ values between 52.13 and 215.14 nM (Table 11). Among the synthesized compounds, **3b** exhibited the most favorable binding profile, with the lowest predicted binding energy of −9.94 kcal/mol and the lowest estimated K_i_ value of 52.13 nM. This was followed by **3d** and **3c**, which showed comparable binding energies of −9.79 and −9.76 kcal/mol, respectively, whereas **3a** displayed a relatively weaker but still favorable binding energy of −9.10 kcal/mol. As expected, the reference PI3Kα inhibitor alpelisib exhibited the strongest predicted binding affinity, with a binding energy of −11.67 kcal/mol and an estimated K_i_ value of 2.79 nM, supporting the internal consistency of the docking protocol.

The superior docking profile of compound **3b** is noteworthy because this derivative also exhibited one of the strongest antiproliferative activities in the in vitro assays. While molecular docking alone cannot establish a direct mechanistic relationship, the agreement between the computational predictions and biological observations suggests that interaction with PI3Kα may represent one of the possible contributors to the observed anticancer activity. Nevertheless, the docking results should be interpreted as hypothesis-generating rather than definitive evidence of PI3Kα inhibition. This agreement between the in vitro and in silico data strengthens the prioritization of **3b** as the most promising compound among the synthesized triazole derivatives. Furthermore, it should be noted that 5-fluorouracil (5-FU) was employed in the biological experiments as a clinically established reference anticancer drug for antiproliferative activity comparison and was not intended to serve as a mechanistic reference compound for PI3Kα inhibition.

**Figure 10:**
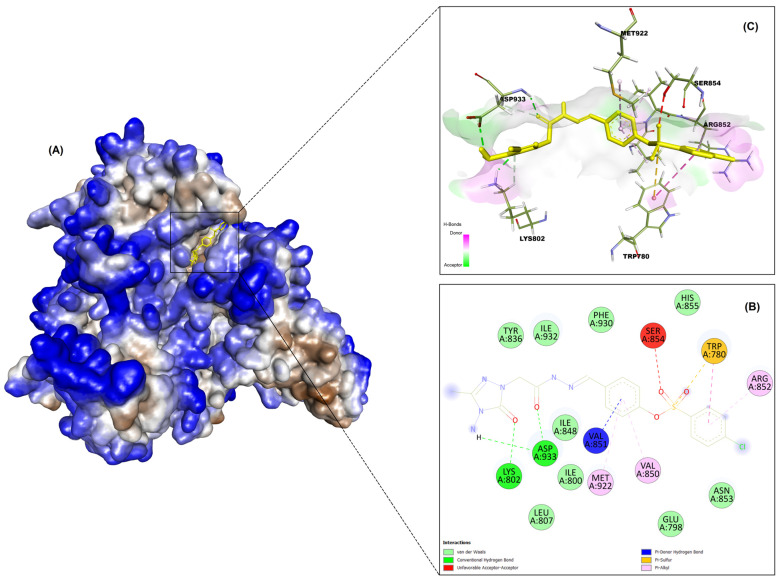
Binding mode of compound **3b** in the PI3Kα ATP-binding pocket. (**A**) Surface view of **3b** within the catalytic cleft. (**B**) 2D interaction map showing key residue contacts. (**C**) 3D close-up view highlighting the spatial orientation and major interactions of **3b**.

The predicted binding orientation of **3b** further supports its favorable docking profile. The surface representation of the PI3Kα–3b complex showed that compound **3b** was well accommodated within the ATP-binding cleft of PI3Kα, occupying the catalytic pocket in a spatially compatible orientation (Figure 10A). The two-dimensional interaction diagram demonstrated that **3b** established multiple stabilizing interactions with key residues, including Trp780, Lys802, Val850, Val851, Arg852, Ser854, Met922, and Asp933 (Figure 10B). These contacts included conventional hydrogen bonds, carbon–hydrogen bonds, π–π stacking, π–alkyl, π–sulfur, and van der Waals interactions, indicating that ligand stabilization was mediated by a balanced contribution of polar and hydrophobic interactions. The three-dimensional interaction view also confirmed the close positioning of **3b** within the binding pocket and highlighted its interaction network with Lys802, Trp780, Val851, Ser854, Met922, and Asp933 (Figure 10C).

Interaction analysis revealed that compounds **3a**–**3d** shared several common contacts with residues located in the PI3Kα ATP-binding region, including Trp780, Lys802, Tyr836, Val850, Val851, Arg852, Ser854, Met922, and Asp933 (Table 11; Figure 10B,C). These residues are known to contribute to ligand recognition and stabilization within the PI3Kα catalytic cleft, particularly through hydrogen bonding, hydrophobic interactions, aromatic contacts, and van der Waals interactions. In PI3Kα inhibitor complexes, interactions with hinge-region and adjacent pocket residues such as Val851, Ser854, Tyr836, Met922, Gln859, Ile932, and Asp933 are considered important for ATP-competitive inhibitor binding and selectivity [49,50]. In the present study, the involvement of Val851 and Ser854 in the binding mode of **3b** is especially notable, as these residues are located near the hinge region and frequently participate in the stabilization of PI3Kα inhibitors within the ATP-binding pocket.

Although compound **3d** formed the highest number of hydrogen bonds among the synthesized derivatives, its predicted binding energy was slightly weaker than that of **3b**. This indicates that the overall binding affinity was not determined solely by the number of hydrogen bonds, but rather by the combined contribution of hydrogen bonding, hydrophobic complementarity, aromatic interactions, ligand orientation, and close-contact interactions within the catalytic cleft. This interpretation is consistent with structure-based drug design principles, where docking scores should be evaluated together with interaction geometry, residue-level contacts, and biological activity rather than being considered as standalone indicators of potency [47,51].

Overall, the docking findings indicate that the synthesized triazole derivatives are capable of establishing favorable interactions within the ATP-binding pocket of PI3Kα and may potentially interact with this therapeutically relevant target. Among them, compound **3b** displayed the most favorable predicted binding energy and Ki value, and this result is consistent with its superior in vitro activity. The interaction profile shown in Figure 10, together with the docking scores summarized in Table 11, supports the prioritization of **3b** as the most promising derivative in this series. The recurrent interactions of **3b** with key PI3Kα residues, particularly Val851, Ser854, Met922, and Asp933, suggest that this compound may adopt a binding mode compatible with known ATP-competitive PI3Kα inhibitors. However, it should be emphasized that the present docking study was designed to explore a potential molecular target associated with the observed biological activity and was not intended to establish PI3Kα inhibition as the sole or definitive mechanism of action. Therefore, compound **3b** may be considered a suitable candidate for further mechanistic validation, including molecular dynamics simulations and binding free-energy analyses.

Although molecular docking suggested favorable interactions between the synthesized derivatives and the PI3Kα catalytic pocket, the present results do not allow direct attribution of the observed antiproliferative activity solely to PI3Kα inhibition. The biological experiments demonstrated that the compounds were capable of interacting with DNA, inducing DNA degradation, suppressing cell migration, and reducing cancer cell viability. These findings indicate that the anticancer activity may arise from multiple complementary mechanisms. DNA-binding and DNA degradation studies provide direct experimental evidence that the synthesized compounds can interact with genetic material and potentially induce DNA damage-related cellular responses. In parallel, the docking results suggest that modulation of PI3Kα-related signaling pathways may also contribute to the observed biological effects. Therefore, rather than supporting a single dominant mechanism, the available data collectively suggest a multifactorial mode of action involving both DNA-directed effects and potential interference with cancer-associated signaling pathways. Consequently, the docking results should be interpreted as evidence supporting a possible contribution of PI3Kα-related signaling to the overall biological response rather than definitive proof of a target-specific mechanism. Further studies involving kinase inhibition assays, pathway analysis, apoptosis markers, and protein expression studies will be required to establish the precise molecular mechanism of action.

### 2.13. In Silico ADME and Toxicity Assessment

To further evaluate the developability potential of the synthesized compounds, preliminary in silico drug-likeness, pharmacokinetic, and toxicity analyses were performed using SwissADME (http://www.swissadme.ch, accessed 1 April 2026), ADMETlab 3.0, and ProTox-3.0. The predicted properties are summarized in Table 12.

The SwissADME analysis indicated that compounds **3a** and **3b** complied with Lipinski’s rule of five with only one violation related to the number of heteroatoms, whereas compounds **3c** and **3d** exhibited two violations due to their higher molecular weights (>500 g/mol) in addition to the elevated heteroatom count. Consistent with their lower molecular weights, compounds **3a** and **3b** also showed higher predicted bioavailability scores (0.55) than compounds **3c** and **3d** (0.17). All compounds displayed low consensus LogP values (0.41–0.78), suggesting relatively low lipophilicity, while the high TPSA values (159.05 Å^2^) were associated with low predicted gastrointestinal absorption and lack of blood–brain barrier permeability.

Pharmacokinetic predictions further indicated that all compounds were likely P-glycoprotein substrates and were not predicted to inhibit CYP3A4 or CYP2D6, suggesting a potentially low risk of major cytochrome P450-mediated drug–drug interactions. Toxicity predictions obtained from ProTox-3.0 classified all derivatives as toxicity class 4 compounds with a predicted oral LD50 value of 500 mg/kg. Furthermore, none of the investigated compounds showed predicted hepatotoxic, mutagenic, or carcinogenic liabilities.

Overall, the ADME and toxicity analyses suggest that the synthesized derivatives possess acceptable preliminary safety profiles, although their relatively high polarity may limit oral absorption. Among the investigated compounds, **3b** demonstrated the most balanced combination of antiproliferative activity, favorable docking performance, and predicted developability-related properties, supporting its prioritization for further biological and mechanistic investigations.

## 3. Materials and Methods

### 3.1. Instruments

The IR spectra of the synthesized compounds were recorded using a Perkin Elmer (Waltham, MA, USA) FT-IR 1600 spectrophotometer in the range of 4000–400 cm^−1^. The ^1^H-NMR and ^13^C-NMR spectra were obtained using a Bruker (Billerica, MA, USA) 400 MHz NMR spectrometer with DMSO-d_6_ as the solvent. Mass spectra were obtained from the Thermo (Waltham, MA, USA) Surveyor Tsq Quantum Access instrument. All solvents and reagents used in the synthesis and structural characterization were purchased from Fluka (Buchs, Switzerland), Merck (Boston, MA, USA), and Aldrich (Saint Louis, MO, USA), and were subjected to appropriate purification and drying procedures before use.

### 3.2. Synthesis of Compounds (***3a***–***3d***)

**Scheme 1:**
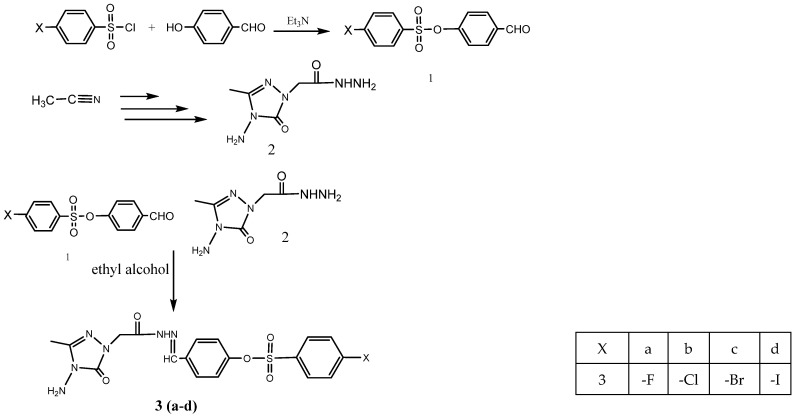
Synthetic pathways of compounds **3a**–**d**.

4-Formylphenyl 4-halogenbenzenesulfonate derivatives (**1a**–**1d**) (**1a** = F, **1b** = Cl, **1c** = Br, and **1d** = I). (1.00 mmol each; 0.280 g, 0.296 g, 0.341 g, and 0.388 g, respectively) and 2-(4-amino-3-methyl-5-oxo-4,5-dihydro-1H-1,2,4-triazol-1-yl)acetohydrazide (2) (0.186 g, 1.00 mmol) were refluxed in equimolar amounts in ethanol for 6 h. The progress of the reaction was monitored by TLC. Upon completion, the reaction mixture was cooled to room temperature, and the solvent was removed under reduced pressure. The resulting solid was purified by recrystallization from an ethanol–water mixture. Compounds **1** and **2** are known compounds reported in the literature and were synthesized according to the procedures described in references [52,53] (Scheme 1).

*(E)-4-((2-(2-(4-amino-3-methyl-5-oxo-4,5-dihydro-1H-1,2,4-triazol-1-yl) acetyl) hydrazono) methyl) phenyl 4-fluorobenzenesulfonate* (**3a**): Yield: 86%, m.p. 262–264 °C. IR (KBr, cm^−1^): 3324, 3269 (NH2), 3106 (NH), 3083 (=CH), 1718, 1692 (C=O), 1625 (C=N), 1592 (C=C); ^1^H NMR (400 MHz, DMSO-d_6_) δ: 2.12 (s, CH_3_, 3H), 4.39, 4.80 (s, N-CH_2_, 2H), 5.32 (s, NH_2_, 2H), Arom H [7.11 (t, 2H), 7.53 (t, 2H), 7.70–7.76 (m, 2H), 7.96 (t, 2H)], 7.96 (t, N=CH,1H), 11.73 (s, NH, 1H); ^13^C NMR (100 Hz, DMSO-d_6_) δ: 11.07 (CH_3_), 46.56 (CH_2_), F-C_6_H_4_ [117.63, 117.76 (CH), 130.72, 130.79 (C), 132.13, 132.23 (CH), 164.85, 167.38 (C)], Arom.C [123.02 (CH), 128.93 (CH), 133.80 (C), 153.99 (C)] 142.85 (N=CH), 145.31 (C=N), 150.09 (triazol C=O), 168.81 (C=O). LC-MS/MS *m*/*z*: [M + Na]^+^ calcd for C_18_H_17_FN_6_O5S, 471.08; found, 470.89.
*(E)-4-((2-(2-(4-amino-3-methyl-5-oxo-4,5-dihydro-1H-1,2,4-triazol-1-yl) acetyl) hydrazono) methyl) phenyl 4-chlorobenzenesulfonate* (**3b**): Yield: 89%, m.p. 258–260 °C. IR (KBr, cm^−1^): 3321, 3274 (NH2), 3102 (NH), 3073 (=CH), 1717,1693 (C=O), 1626 (C=N), 1588 (C=C); ^1^H NMR (400 MHz, DMSO-d_6_) δ: 2.12 (s, CH_3_, 3H), 4.39, 4.80 (s, N-CH_2_, 2H), 5.31 (s, NH_2_, 2H), Arom H [7.12 (bs, 2H), 7.76–7.96 (m, 6H)], 7.76–7.96 (m, N=CH,1H), 11.72 (s, NH, 1H); ^13^C NMR (100 Hz, DMSO-d_6_) δ: 11.10 (CH_3_), 46.55 (CH_2_), Cl-C_6_H_4_ [130.56 (CH), 130.72 (CH), 130.80 (C), 140.66 (C)], Arom.C [123.07 (CH), 129.10 (CH), 133.80 (C), 154.04 (C)] 142.83 (N=CH), 145.30 (C=N), 150.04 (triazol C=O), 168.74 (C=O). LC-MS/MS *m*/*z*: [M + Na]^+^ calcd for C_18_H_17_ClN_6_O5S, 487.05; found, 486.94.
*(E)-4-((2-(2-(4-amino-3-methyl-5-oxo-4,5-dihydro-1H-1,2,4-triazol-1-yl) acetyl) hydrazono) methyl) phenyl 4-bromobenzenesulfonate* (**3c**): Yield: 75%, m.p. 256–258 °C. IR (KBr, cm^−1^): 3320, 3277 (NH2), 3095 (NH), 3073 (=CH), 1720,1695 (C=O), 1608 (C=N), 1574 (C=C); ^1^H NMR (400 MHz, DMSO-d_6_) δ: 2.12 (s, CH_3_, 3H), 4.39, 4.80 (s, N-CH_2_, 2H), 5.31 (s, NH_2_, 2H), Arom H [7.10–7.14 (m, 2H), 7.71–7.96 (m, 6H)], 7.71–7.96 (m, N=CH,1H), 11.72 (s, NH, 1H); ^13^C NMR (100 Hz, DMSO-d_6_) δ: 11.13 (CH_3_), 46.66 (CH_2_), Br-C_6_H_4_ [130.68 (CH), 133.51 (CH), 133.73 (C), 133.86 (C)], Arom.C [123.02 (CH), 129.23 (CH), 133.86 (C), 154.08 (C)] 142.83 (N=CH), 145.44 (C=N), 150.13 (triazol C=O), 168.90 (C=O). LC-MS/MS *m*/*z*: [M + 1]^+^ calcd for C_18_H_17_BrN_6_O5S, 531.00; found, 530.85.
*(E)-4-((2-(2-(4-amino-3-methyl-5-oxo-4,5-dihydro-1H-1,2,4-triazol-1-yl) acetyl) hydrazono) methyl) phenyl 4-iodobenzenesulfonate* (**3d**): Yield: 90.55%, m.p. 270–272 °C. IR (KBr, cm^−1^): 3321, 3279 (NH_2_), 3109 (NH), 3086 (=CH), 1720, 1695 (C=O), 1609 (C=N), 1566 (C=C); ^1^H NMR (400 MHz, DMSO-d_6_) δ: 2.12 (s, CH_3_, 3H), 4.39, 4.80 (s, N-CH_2_, 2H), 5.31 (s, NH_2_, 2H), Arom H [7.12 (bs, 2H), 7.60–8.18 (m, 6H)], 7.60–8.18 (m, N=CH,1H), 11.72 (s, NH, 1H); ^13^C NMR (100 Hz, DMSO-d_6_) δ: 11.13 (CH_3_), 46.66 (CH_2_), I-C_6_H_4_ [104.99 (C), 130.15 (CH), 134.20 (C), 139.27 (CH)], Arom.C [122.84 (CH), 129.16 (CH), 133.76 (C), 153.88 (C)] 142.85 (N=CH), 145.22 (C=N), 150.06 (triazol C=O), 168.77 (C=O). LC-MS/MS *m*/*z*: [M + Na]^+^ calcd for C_18_H_17_IN_6_O5S, 578.99; found, 578.87.

### 3.3. Computational Methods

All DFT calculations of compounds (**3a**–**3d**) were performed with the DFT/B3LYP method and LANL2DZ [54] basis set in the Gaussian 09 [55] package program. The GaussView [56] program was used to visualize the results obtained from the calculations. A scale factor of 0.987 was used to ensure the agreement of the values of the vibration frequencies obtained from the calculations with the experimental vibration frequencies. GIAO [57,58] approach was used for chemical shift calculations of the molecules. ^1^H and ^13^C calculations were determined using scale factors of 32.75 and 193.73 ppm, respectively.

### 3.4. Cell Lines and Cell Culture

Human glioblastoma cell lines A172 (ATCC CRL-1620), U87MG (ATCC HTB-14), and IDHmut-U87 (ATCC HTB-14IG), rat glioma cell line C6 (ATCC CCL-107), human neuroblastoma cell line SH-SY5Y (ATCC CRL-2266), and rat neuroblastoma cell line B35 (ATCC CRL-2754), human colorectal adenocarcinoma cells HT29 (ATCC HTB-38), human cervical adenocarcinoma cells HeLa (ATCC CCL2), human breast adenocarcinoma cells MCF7 (ATCC HTB22), and human normal cells FL (ATCC CCL-62) were used in this study. All cell culture procedures were performed under sterile conditions in a laminar flow cabinet. Cells were cultured in DMEM or RPMI-1640 medium supplemented with 10% fetal bovine serum (FBS) and 2% penicillin–streptomycin, and maintained at 37 °C in a humidified atmosphere containing 5% CO_2_. For experiments, cells were seeded at a density of 1 × 10^4^ cells per well in measurement plates. After approximately 16 h of preincubation to allow cell attachment, test compounds were added, and analyses were conducted following an additional 24 h incubation period.

### 3.5. Measurement of Cell Proliferation

The MTT assay was used to evaluate the effects of the test molecules on cell proliferation following 24 h of incubation with cancer cell lines. Results were expressed as percentage inhibition, with the optical density of solvent-treated (DMSO) cells defined as 100%. Percentage inhibition was calculated using the formula: % inhibition = [1 − (A test/A control)] × 100. IC_50_ values, defined as the concentration required to inhibit cell proliferation by 50%, were determined from absorbance values obtained after treatment with increasing concentrations of each compound (1.96–125.0 µg/mL). Dose–response curves were generated using logarithmic regression analysis in Microsoft Excel. GI_50_ values were calculated based on cell growth according to the following equations: [(Ti − Tz)/(C − Tz)] × 100 for Ti ≥ Tz (cytostatic effect) or [(Ti − Tz)/Tz] × 100 for Ti < Tz (cytotoxic effect), where Tz represents the absorbance at time zero, C denotes control growth, and Ti indicates growth in the presence of the test compound. GI_50_ was defined as the concentration producing 50% growth inhibition. Tumor selectivity index (TSI) values were calculated as the ratio of the IC_50_ concentration in normal cells to the mean IC_50_ concentration in cancer cells.

### 3.6. Cytotoxicity Test

Cytotoxic and cytostatic effects of the test molecules were evaluated using the lactate dehydrogenase (LDH) release assay. LDH is a stable cytoplasmic enzyme that is released into the culture supernatant upon damage to the plasma membrane, and increased LDH levels therefore reflect enhanced cell death. The LDH cytotoxicity assay was performed in accordance with the manufacturer’s instructions. Formazan formation resulting from LDH enzymatic activity was quantified spectrophotometrically, and percentage cytotoxicity was calculated using the following equation:

% Cytotoxicity = [(A sample − A low control)/(A high control − A low control)] × 100.

### 3.7. DNA Fragmentation by Agarose Gel Electrophoresis

The DNA laddering activity of the compounds was evaluated using a DNA laddering assay according to the standard method. Briefly, 7.5 × 10^5^ cells were placed into 25 cm^2^ culture flasks and treated with IC_50_ concentrations of the compounds at 37 °C with 5% CO_2_ overnight. First, DNA-containing precipitate was extracted from the digest using 50 µL of phosphate-citrate buffer and centrifuged at 1500× *g* for 5 min. Then, 40 µL of the supernatant was transferred to a microcentrifuge tube. A solution of Tween 20 (5 µL) (0.25% in ddH_2_O) and RNase A (5 µL) was added to the supernatant and incubated at 37 °C for 30 min. Next, proteinase K (5 µL) was added to each tube, and the mixture was re-incubated at 37 °C for 5 min. Finally, the DNA-containing precipitate from the microcentrifuge tube was combined with 5 µL of loading buffer and loaded onto a 1.5% agarose gel containing 0.5 µg/mL ethidium bromide, which was then electrophoresed at 200 mA for 40 min. After electrophoresis, DNA fragmentation on the gels was imaged using a UVP gel imaging system.

### 3.8. Migration Assay

Cell migration was evaluated using an in vitro wound-healing (scratch) assay. Briefly, a linear scratch was created across the confluent cell monolayer using a sterile p200 pipette tip. Detached cells and debris were removed by washing with 1 mL of growth medium to ensure clean wound edges. Fresh medium was then added, and test compounds were applied at their respective GI_50_ concentrations. Images of the marked wound area were captured immediately after treatment and subsequently at 24 h intervals. Wound closure was monitored until the scratch gap of approximately 500 μm in the untreated control was completely closed, allowing quantitative assessment of cell migration.

### 3.9. Determination of DNA Binding Constant

The UV/Vis absorption method was used for the DNA binding assay. Experiments investigating the interactions of CT-DNA molecules were conducted in a buffer solution containing 5 mM Tris and 50 mM NaCl, with the pH adjusted to 7.3 using HCl. Stock solutions of the test molecules were prepared at a concentration of 1 × 10^−3^ M in DMSO. Absorption titration experiments were performed based on the principle of maintaining a constant ligand concentration while gradually increasing the concentration of CT-DNA. Based on the results obtained, the [DNA]/(εa − εf) values were plotted against the corresponding DNA concentrations ([DNA]) using the Wolfe–Shimmer equation. The slope of the line obtained from the graph yields 1/(εb − εf), while the intercept point provides 1/Kb(εb − εf). The binding constants, Kb, were calculated by dividing these two values. In this equation, εf represents the molar absorption coefficient of the free compound, εb denotes the molar absorption coefficient of the fully bound compound, and εa is A/[M]. Wolfe–Shimmer equation: [DNA]/(|εa − εF|) = [DNA]/(|εB − εF|) + 1/{Kb (|εB − εF|)}.

### 3.10. Antimicrobial Activity

The newly synthesized compounds underwent assessment for their antimicrobial properties against nine distinct pathogenic microorganisms responsible for various diseases. This selection encompassed eight microorganisms, encompassing both Gram-negative bacteria (*Escherichia coli* ATCC 25922, *Yersinia pseudotuberculosis* ATCC 911, *Pseudomonas aeruginosa* ATCC 17853, *Klebsiella pneumoniae* ATCC 700603) and Gram-positive bacteria (*Staphylococcus aureus* ATCC 25923, *Bacillus subtilis* ATCC 6633, *Bacillus cereus* ATCC 10876, *Enterococcus faecalis* ATCC 29212), as well as yeast-like fungi (*Candida albicans* ATCC 10231, *Candida tropicalis* ATCC 13803). These microorganisms were procured from the American Type Culture Collection.

The minimal inhibitory concentration (MIC) values, expressed in micrograms per milliliter (μg/mL), of the newly synthesized compounds were ascertained utilizing the microtitre broth dilution method, accompanied by the rapid INT colorimetric assay as detailed in Kuete et al. [59], following the guidelines established by the Clinical and Laboratory Standards Institute (CLSI). To prepare stock solutions of the synthesized compounds, all compounds were dissolved in dimethyl sulfoxide (DMSO). These stock concentrations were then subjected to a two-fold serial dilution in 96-well microplates using Mueller–Hinton broth (MHB) as the diluent. Bacterial suspensions were prepared at a concentration of approximately 5 × 10^−5^ colony-forming units (CFU) per milliliter and were subsequently inoculated into each well. The microplates were then incubated at 37 °C for a period of 24 h. After the 37 °C incubation, each well received an addition of 40 μL of a 0.2 mg/mL solution of p-iodonitrotetrazolium chloride (INT), serving as an indicator of microbial growth. The microplates were further incubated for 30 min at 37 °C, during which the MIC values were visually determined. The MIC value was recognized as the lowest concentration at which the growth of the tested microorganism was entirely inhibited, which corresponded to the appearance of the first clear well. MIC values were determined at least in triplicate, with the value being considered the final MIC when it was repeated at least two times. Ampicillin (10 mg/mL) and fluconazole (2 mg/mL) were used as standard commercial drugs for bacterial and yeast testing, respectively. A negative control experiment was conducted, employing only DMSO to account for any inhibitory effects stemming from the solvent.

### 3.11. Molecular Docking Studies

Molecular docking is a widely used structure-based computational approach that predicts the preferred orientation of small molecules within the active site of a biological macromolecule and estimates their binding affinity. It plays a critical role in modern drug discovery by enabling mechanistic interpretation of in vitro findings and prioritization of promising candidates prior to advanced experimental validation [47,51]. The phosphatidylinositol-3-kinase (PI3K)/Akt signaling pathway plays a pivotal role in the regulation of cancer cell proliferation, survival, migration, and resistance to apoptosis. Aberrant activation of PI3Kα has been reported in several cancer types investigated in the present study, including glioblastoma, neuroblastoma, breast cancer, and colorectal cancer. Moreover, structurally related triazole- and hydrazone-containing compounds have previously been reported to exert anticancer effects through modulation of PI3K/Akt-associated signaling pathways. Therefore, PI3Kα was selected as a biologically relevant exploratory target to investigate potential ligand–target interactions and provide preliminary mechanistic insights into the observed antiproliferative activity. In the present study, molecular docking was performed to investigate the binding potential of newly synthesized triazole derivatives (**3a**–**3d**) toward phosphatidylinositol-4,5-bisphosphate 3-kinase catalytic subunit alpha (PI3Kα), a key enzyme in the PI3K/AKT signaling pathway frequently dysregulated in lung and breast cancer. The docking analysis was intended to explore a possible target associated with the observed biological activity rather than to establish PI3Kα inhibition as the definitive mechanism of action.

The X-ray crystal structure of human PI3Kα (PDB ID: 4JPS) was retrieved from the RCSB Protein Data Bank. Only the catalytic subunit (Chain A; p110α) was retained for docking simulations, whereas the regulatory subunit (Chain B) was removed. Protein preparation was performed using UCSF ChimeraX 1.6 [60]. Crystallographic water molecules and non-essential heteroatoms were deleted, missing residues were modeled, protonation states were assigned assuming physiological pH conditions, and the structure was locally energy-minimized to eliminate steric clashes while preserving the crystallographic geometry. The co-crystallized inhibitor was used to define the ATP-binding region and for protocol validation, and subsequently removed prior to docking. Kollman charges and polar hydrogens were added using AutoDockTools 1.5.7 [48], and the receptor was saved in PDBQT format.

Ligands **3a**–**3d** were drawn in ChemDraw Professional 22.0 (PerkinElmer Informatics) and converted into three-dimensional structures. Geometry optimization was carried out using Avogadro 1.97 [61] with the MMFF94 force field and steepest descent algorithm. Protonation states and potential tautomeric forms were evaluated under physiological pH conditions to ensure chemically relevant docking inputs. Gasteiger–Marsili charges were assigned, and rotatable bonds were defined in AutoDockTools prior to PDBQT conversion.

Docking grid maps were generated using AutoGrid 4.2 and centered on the ATP-binding pocket of PI3Kα using the geometric coordinates of the co-crystallized inhibitor (grid center: x = −1.96, y = −11.02, z = 16.31). The grid box dimensions were defined as 60 × 60 × 60 points with a grid spacing of 0.375 Å, ensuring full coverage of the catalytic cleft and adjacent subpockets relevant for ATP-competitive inhibition. Docking simulations were carried out using AutoDock 4.2.6 implementing the Lamarckian Genetic Algorithm (LGA), with 150 independent runs per ligand. The population size was set to 150 individuals, with a maximum of 2.5 × 10^6^ energy evaluations and 27,000 generations per run. All remaining parameters were kept at default values. Resulting conformations were clustered using a 2.0 Å RMSD tolerance, and the representative pose from the most populated cluster exhibiting the lowest predicted binding free energy was selected for further structural analysis.

The docking protocol was validated by re-docking the co-crystallized inhibitor under identical parameters. Superposition of the predicted and crystallographic poses yielded an RMSD ≤ 2.0 Å, confirming the robustness of the docking setup. Protein–ligand interactions, including hydrogen bonds, π–π stacking, hydrophobic contacts, and electrostatic interactions, were analyzed and visualized using BIOVIA (San Diego, CA, USA) Discovery Studio Visualizer 2021 [62].

### 3.12. In Silico ADME and Toxicity Prediction

To obtain preliminary information regarding the drug-likeness, pharmacokinetic behavior, and toxicity profiles of the synthesized compounds (**3a**–**3d**), in silico ADME and toxicity analyses were performed using the SwissADME, ADMETlab 3.0, and ProTox web servers [63,64,65]. Canonical SMILES representations of the compounds were generated and used as input structures for all calculations.

SwissADME was employed to evaluate physicochemical properties, Lipinski’s rule of five compliance, gastrointestinal (GI) absorption, blood–brain barrier (BBB) permeability, and medicinal chemistry friendliness parameters [13]. ADMETlab 3.0 was used to predict absorption, distribution, metabolism, and excretion (ADME)-related properties, including human intestinal absorption (HIA), P-glycoprotein interactions, and cytochrome P450-associated parameters [64]. Toxicological properties, including predicted oral LD50 values and toxicity classes, were estimated using the ProTox platform [15]. The obtained results were comparatively analyzed to assess the potential developability of the synthesized derivatives.

## 4. Conclusions

A new series of (E)-4-((2-(2-(4-amino-3-methyl-5-oxo-4,5-dihydro-1H-1,2,4-triazol-1-yl)acetyl)hydrazono)methyl)phenyl 4-halogenobenzenesulfonates (**3a**–**3d**), where **3a** = F, **3b** = Cl, **3c** = Br, and **3d** = I, were successfully synthesized via a straightforward synthetic route. The structures of the obtained compounds were fully characterized and confirmed by spectroscopic techniques, including FT-IR, ^1^H NMR, and ^13^C NMR, as well as LC-MS/MS analysis. In this study, molecules **3a**–**3d** were analyzed using experimental and quantum mechanical methods. In this work, the structural parameters, vibrational properties, frontier molecular orbital energies, and electronic properties of these molecules were investigated. The molecules were characterized by ^1^H NMR, ^13^C NMR, and FT-IR techniques. The HOMO and LUMO energies of the molecules were used to determine chemical hardness, chemical softness, electronegativity, and electronic structure. Based on the MEP surface, it was found that the most reactive region of the compound for electrophilic attack is around the nitrogen and oxygen atoms, while the most reactive region for nucleophilic attack was determined to be around the NH in the triazole ring. Important conclusions were obtained regarding charge flow in the molecules. Additionally, dipole moment, polar, and hyperpolar parameters were calculated, and the molecules were found to possess NLO properties. These molecules were found to have important characteristics for application purposes when utilized in further studies. The present study demonstrated that compounds **3a**–**3d** possess significant anticancer potential through multiple interconnected biological mechanisms. The compounds exhibited notable antiproliferative effects in various cancer cell lines while generally producing relatively low membrane toxicity compared with the reference drug 5FU. DNA-binding studies revealed moderate interactions with DNA, suggesting that DNA associations may contribute to their biological activity. Furthermore, substantial DNA degradation was observed, particularly in C6 and SH-SY5Y cells, supporting the involvement of apoptosis-associated processes. Migration analysis showed reduced wound closure, indicating potential anti-migratory and anti-invasive properties. Morphological examinations further confirmed treatment-induced cellular alterations, including cell shrinkage, rounding, detachment, and loss of normal architecture. Overall, these findings suggest that the tested compounds may represent promising multifunctional anticancer candidates. However, additional mechanistic studies are required to further clarify their molecular targets and therapeutic potential. In addition, the synthesized compounds **3a**–**3d** exhibited moderate antimicrobial and antifungal activities against the tested microorganisms. Although their inhibitory effects were lower than those of the reference drugs, the results demonstrate that the triazole–hydrazone-based scaffold possesses measurable biological potential. These findings may serve as a basis for further structural optimization studies aimed at enhancing antimicrobial efficacy. The molecular docking results indicated that the synthesized triazole derivatives exhibited favorable binding profiles within the ATP-binding pocket of PI3Kα. Among the tested compounds, **3b** showed the strongest predicted binding affinity, the lowest estimated Ki value, and stable interactions with key residues such as Val851, Ser854, Met922, and Asp933. The agreement between the docking results and the superior in vitro activity of **3b** supports the biological relevance of its predicted binding mode. However, considering the DNA-binding and DNA degradation results, the observed anticancer activity is likely associated with multiple complementary mechanisms rather than solely PI3Kα modulation. Overall, these findings suggest that **3b** is the most promising derivative in this series and may be considered for further mechanistic validation through molecular dynamics simulations, binding free-energy calculations, and additional experimental studies. Furthermore, preliminary in silico ADME and toxicity analyses indicated acceptable drug-likeness characteristics, absence of predicted hepatotoxic, mutagenic, and carcinogenic liabilities, and moderate acute toxicity profiles, supporting the developability potential of the synthesized compounds.

## Figures and Tables

**Table 1:** Selected molecular structure parameters of the compounds.

Vibrations	3a (Exp.)	3b (Exp.)	3c (Exp.)	3d (Exp.)
ν (NH_2_) as	3592 (3324)	3592 (3321)	3592 (3320)	3592 (3321)
ν (NH_2_) s	3466 (3269)	3466 (3274)	3466 (3277)	3467 (3279)
ν (N-H) s	3384 (3106)	3380 (3102)	3384 (3095)	3387 (3109)
ν (=CH)s R	3204 (3083)	3205 (3073)	3208 (3073)	3205 (3086)
ν (=CH) as R	3183	3188	3188	3185
ν (C-H_2_) as	3155	3155	3155	3154
ν (C-H) al	3151	3151	3151	3151
ν (C-H_3_) as	3143	3143	3143	3143
ν (C-H_2_) s	3034	3033	3034	3034
ν (C-H_3_) s	3024	3024	3024	3024
α (N-H2)	1696	1696	1696	1696
ν (C=O) + γ (N-H)	1680 (1718)	1680 (1717)	1680 (1720)	1680 (1720)
ν (C=O) + γ (N-H)	1646 (1692)	1646 (1693)	1646 (1695)	1646 (1695)
ν (C=N) s + ν (C=O)	1595 (1625)	1595 (1626)	1595 (1608)	1595 (1609)
ν (C=C) R	1587 (1592)	1592 (1588)	1587 (1574)	1592 (1566)
ν (C-N) tri.	1584	1584	1580	1584
γ (C-N) + ν (C=N) s	1546	1545	1546	1546
γ (CH)	1492	1479	1477	1475
ω (C-N_2_) + α (C-H_2_)	1473	1472	1472	1473
ν (N-NH_2_) + ω (C-H_3_)	1441	1441	1442	1441
α (C-H) R	1394	1403	1399	1395
ν (N-CH_2_) + ω (C-H_2_)	1382	1382	1381	1382
β (CCC) R1, R2	1371	1353	1349	1349
β (CCC) R1, R2	1349	1349	1347	1342
ν (S-O_2_) as	1290	1311	1291	1291
ν (NH-CO)	1273	1273	1272	1273
Q, R1, R2 + ν (NH-CO) + ν (F-C)	1216	1243	1243	1243
ν (C-O) + α (C-H)	1158	1158	1158	1158
ν (N-N) tri + ν (N-NH)	1095	1096	1095	1095
Q, R1	1062	1065	1057	1049
δ (C-H) R1, R2	1025	1025	1024	1024
δ (C-H) R1	1016	1006	1010	1010
β (CCC) R1 + ν (Br-C) + ν (S-C)	1011	1003	998	995
ν (S-O_2_) as	944	944	944	944
ω (N-H)	898	898	898	894
ω (C-H) R2	889	890	890	889
ω (C-H) R1	873	865	866	865
ν (C-SO_2_) as + ν (S-O_2_) as	827	825	825	825
ν (C-O-S)	806	806	806	806
τ (OCN) tri. + ω (N-H_2_)	730	730	730	730
τ (CCC) R1 ν (Cl-C)	727	724	722	721
β (CCC) R1 + ν (Br-C) + ν (Cl-C)	628	712	701	695
β (NCN) tri.	601	598	598	604
τ (CCC) R1	535	533	531	521
ν (C-S) + ν (Cl(Br)(I)-C)	472	507	491	487
ν (O-S)	456	462	456	454
ν (X-C)	412	379	342	332

ν, stretching; α, scissoring; γ, rocking; ω, wagging; δ, twisting; Q, breathing; β, in-plane bending; τ, out-of-plane bending; s, symmetric; as, asymmetric; al, aliphatic, tri, triazole. Abbreviations: X: F, Cl, Br, I; R1 (C21-C26) and R2 (C11-C16); R, R1, R2 rings.

**Table 2:** Theoretical and experimental scaled ^1^H NMR data for compounds.

Atom	3a (T/E)	3b (T/E)	3c (T/E)	3d (T/E)
H31	2.28/2.12	2.27/2.12	2.27/2.12	2.28/2.12
H32	2.48/2.12	2.47/2.12	2.48/2.12	2.47/2.12
H33	2.44/2.12	2.45/2.12	2.46/2.12	2.45/2.12
H34	4.79/4.80	4.79/4.80	4.79/4.80	4.79/4.80
H35	4.31/4.39	4.3/4.39	4.29/4.39	4.29/4.39
H36	11.6/11.73	11.66/11.72	11.64/11.72	11.58/11.72
H37	10.35/7.96	10.34/7.76–7.96	10.34/7.71–7.96	10.33/7.60–8.18
H38	8.82/7.70–7.76	8.81/7.76–7.96	8.81/7.71–7.96	8.8/7.60–8.18
H39	7.9/7.11	7.88/7.12	7.9/7.10–7.14	7.88/7.12
H40	8.02/7.11	7.97/7.12	7.97/7.10–7.14	7.98/7.12
H41	8.03/7.53	8.01/7.76–7.96	8.03/7.71–7.96	8.02/7.60–8.18
H42	8.85/7.96	8.65/7.76–7.96	8.61/7.71–7.96	8.54/7.60–8.18
H43	8.22/7.70–7.76	8.28/7.76–7.96	8.42/7.71–7.96	8.48/7.60–8.18
H45	8.18/7.53	8.21/7.76–7.96	8.4/7.71–7.96	8.44/7.60–8.18
H46	8.82/7.96	8.62/7.76–7.96	8.58/7.71–7.96	8.52/7.60–8.18
H47	4.2/5.32	4.2/5.31	4.21/5.31	4.17/5.31
H48	4.21/5.32	4.2/5.31	4.19/5.31	4.18/5.31

T: Theoretical, E: Experimental.

**Table 3:** Theoretical and experimental scaled ^13^C NMR data for compounds.

Atom	3a (T/E)	3b (T/E)	3c (T/E)	3d (T/E)
C1	16.46/11.07	16.49/11.10	16.36/11.13	16.21/11.13
C2	161.98/145.31	161.49/145.30	161.53/145.44	161.88/145.22
C5	63.47/46.56	63.69/46.55	63.69/46.66	63.51/46.66
C6	183.11/168.81	183.24/168.74	183.11/168.90	183.19/168.77
C10	162.76/142.85	162.71/142.83	162.7/142.83	162.82/142.85
C11	143.82/133.80	143.89/133.80	143.79/133.86	143.67/133.76
C12	130.28/123.02	130.35/123.07	130.27/123.02	130.2/122.84
C13	133.44/123.02	133.36/123.07	133.54/123.02	133.47/122.84
C14	164.46/153.99	164.42/154.04	164.37/154.08	164.36/153.88
C15	134.07/128.93	134.17/129.10	133.92/129.23	133.92/129.16
C16	139.16/128.93	139.05/129.10	139.06/129.23	139.12/129.16
C21	162.18/130.79	164.53/140.66	165.38/133.73	165.82/134.20
C22	131.94/132.23	130.55/130.56	130.22/133.51	129.36/130.15
C23	124.51/117.63	135.32/130.72	139.16/130.68	142.98/139.27
C24	176.72/167.38	161.01/130.80	160.86/133.86	160.02/104.99
C25	124.54/117.76	135.68/130.72	139.27/130.68	142.88/139.27
C26	131.86/132.13	130.76/130.56	130.37/133.51	129.32/130.15
C27	167.61/150.09	167.5/150.04	167.58/150.13	167.58/150.06

T: Theoretical, E: Experimental.

**Table 4:** Quantum chemical parameters calculated at B3LYP/LANL2DZ.

Parameters (eV)	3a	3b	3c	3d
E_LUMO_	−3.31	−3.32	−3.3	−3.27
E_HOMO_	−6.28	−6.27	−6.26	−6.24
ΔE = E_LUMO_ − E_HOMO_	2.96	2.95	2.96	2.97
I = −E_HOMO_	6.28	6.27	6.26	6.24
A = −E_LUMO_	3.31	3.32	3.3	3.27
η = (I − A)/2	1.48	1.47	1.48	1.49
S = 1/2η	0.34	0.34	0.34	0.34
Electronegativity (χ = (I + A)/2)	4.79	4.8	4.78	4.76
Chemical potential (µ = −χ)	−4.79	−4.8	−4.78	−4.76
W = µ2/2η	7.76	7.81	7.7	7.61
ΔN_max_ = µ/η	3.24	3.26	3.23	3.19

**Table 5:** The dipol moment, polarizability, and first hyperpolarizability values.

	3a	3b	3c	3d		3a	3b	3c	3d
Dipol					HyperP.				
μ_x_	−2.006	1.912	1.645	1.381	β_xxx_	13,015.522	−13,280.364	−13,026.568	−12,652.190
μ_y_	1.879	1.990	2.299	2.444	β_xxy_	491.277	113.593	−697.897	−1293.818
μ_z_	0.904	−0.756	−0.518	−0.377	β_xyy_	−139.243	227.865	303.951	310.846
μ_T_	2.893	2.861	2.874	2.833	β_yyy_	9.989	−24.848	−38.940	−47.827
Polar					β_xxz_	−1062.428	1497.343	1571.631	1689.958
α_xx_	580.255	612.519	630.136	651.971	β_xyz_	−83.444	−108.790	−36.142	12.613
α_xy_	24.262	−27.099	−21.149	−19.005	β_yyz_	−4.454	22.424	25.117	28.373
α_yy_	237.744	236.566	233.433	227.083	β_xzz_	−38.635	39.174	−13.366	−16.616
α_xz_	−1.028	−1.499	−7.600	−9.796	β_yzz_	−9.584	−20.981	−6.899	−6.691
α_yz_	−35.887	36.564	37.365	36.175	β_zzz_	58.043	−34.164	−37.631	−36.233
α_zz_	197.074	207.839	216.922	228.357	β_T(esu)_	115.847 × 10^−30^	112.527 × 10^−30^	116.723 × 10^−30^	118.680 × 10^−30^
α_T(esu)_	50.145 × 10^−24^	52.212 × 10^−24^	53.376× 10^−24^	54.706 × 10^−24^	Δα_(esu)_	4.027 × 10^−24^	57.962 × 10^−24^	60.052 × 10^−24^	62.874 × 10^−24^

**Table 6:** GI_50_ and IC_50_ values of substances in brain cancer cell lines.

*Compound* (µM)	A172		C6		SH-SY5Y		B35		U87MG	
	GI_50_	IC_50_	GI_50_	IC_50_	GI_50_	IC_50_	GI_50_	IC_50_	GI_50_	IC_50_
**3a**	20.09	365.74	283.30	410.59	28.10	374.77	226.39	377.25	371.12	384.43
**3b**	174.60	348.56	33.43	303.39	7.59	232.71	>1075.55	377.60	13.85	287.69
**3c**	>981.66	397.91	44.51	276.04	7.62	323.73	>981.66	340.81	508.89	350.55
**3d**	>898.73	346.01	76.86	285.81	435.47	449.04	>898.73	418.09	67.80	376.42
**5FU**	16.68	448.95	14.84	320.34	8.61	155.21	11.99	468.94	14.22	439.81

**Table 7:** GI_50_ and IC_50_ values of substances in brain, colon, cervix, and breast cancer cells and FL normal cells.

*Compound* (µM)	IDHmut-U87		HT29		HeLa		MCF7		FL	
	GI_50_	IC_50_	GI_50_	IC_50_	GI_50_	IC_50_	GI_50_	IC_50_	GI_50_	IC_50_
**3a**	8.16	233.77	7.60	324.26	122.83	300.14	5.60	162.61	11.60	295.81
**3b**	10.56	361.58	5.70	307.37	169.76	322.02	9.85	180.89	62.02	310.94
**3c**	3.87	157.09	3.71	220.87	15.57	218.42	29.31	185.46	6.13	268.92
**3d**	3.27	134.61	22.81	285.58	39.71	260.74	11.20	170.45	4.96	204.61
**5FU**	14.15	293.67	10.99	485.85	14.38	400.37	9.07	367.85	8.61	155.21

**Table 8:** % Cytotoxicity values of the molecules at IC_50_ concentrations.

Compound	A172	C6	SH-SY5Y	B35	U87MG	IDHmut-U87	HT29	HeLa	MCF7	FL
**3a**	3.33	5.77	6.78	6.73	6.63	6.16	4.18	4.60	12.31	6.63
**3b**	6.82	4.19	4.94	5.31	6.36	4.74	3.08	6.74	6.34	6.72
**3c**	4.28	6.56	5.57	3.91	6.22	8.04	6.57	6.47	11.99	3.31
**3d**	4.31	3.72	6.16	4.52	3.28	8.52	5.68	6.32	12.44	4.18
**5FU**	10.12	11.87	14.99	8.33	6.61	13.42	12.10	9.16	12.62	12.70

**Table 9:** Results from the migration analysis of molecules conducted with ImageR.

% Area	C6 Cell Line			SH-SY5Y Cell Line			
	Gap Day 1	Gap Day 3	Gap Filling Ratio	% Area	Gap Day 1	Gap Day 3	Gap Filling Ratio
**3a**	44.21	11.32	32.88	**3a**	51.20	30.95	20.24
**3b**	44.51	9.38	35.13	**3b**	52.12	12.27	39.84
**3c**	41.85	17.15	24.71	**3c**	51.97	19.43	32.54
**3d**	46.53	16.68	29.86	**3d**	57.92	23.78	34.14
**Control**	52.25	0.00	52.25	**Control**	51.88	0.00	51.88

**Table 10:** MIC values of compounds **3a**-d and antibiotics against tested microorganisms.

		Compounds			Antibiotics	
Microorganisms	3a	3b	3c	3d	Ampicillin	Fluconazole
*B. cereus*	156	156	156	156	15	N.T
*B. subtilis*	312	312	312	312	10	N.T
*Y. pseudotuberculosis*	312	625	625	312	18	N.T
*K. pneumoniae*	156	156	156	156	16	N.T
*E. coli*	312	312	312	312	8	N.T
*P. aeruginosa*	312	312	312	312	128	N.T
*S. aureus*	625	312	625	625	10	N.T
*E. faecalis*	312	312	625	312	10	N.T
*C. albicans*	156	312	312	156	N.T	8
*C. tropicalis*	312	156	156	156	N.T	8

Minimal inhibition concentration (MIC) values were given as μg/mL. N.T: not tested.

**Table 11:** Molecular docking results of newly synthesized triazole derivatives (**3a**–**3d**) and reference molecule against PI3Kα.

Ligand	Free Binding Energy (kcal/mol)	Predicted K_i_ Value	Number of Close-Contact Residues	No of H-Bonds	Interacting Key Residues
**3a**	−9.10	215.14 nM	9	4	Trp780, Lys802, Tyr836, Val850, Ser854, Arg852, His855, Met922, Asp933
**3b**	−9.94	52.13 nM	9	4	Trp780, Lys802, Val850, Val851, Arg852, Ser854, Met922, Asp933
**3c**	−9.76	70.45 nM	6	4	Ser774, Trp780, Lys802, Tyr836, Val850, Val851, Arg852, Ser854, His855, Met922, Asp933
**3d**	−9.79	66.71 nM	7	5	Trp780, Lys802, Tyr836, Val850, Val851, Arg852, Ser854, Met922, Asp933
* Alpelisib	−11.67	2.79 nM	7	8	Ile800, Lys802, Tyr836, Ile848, Val850,Val851, Ser854, His855, Gln859, Met922, Ile932

* reference molecule.

**Table 12:** Predicted drug-likeness, pharmacokinetic, and toxicity properties of compounds **3a**–**3d** based on SwissADME, ADMETlab 3.0, and ProTox-3.0 analyses.

Parameter	3a	3b	3c	3d
Molecular Weight (g/mol)	448.43	464.88	509.33	556.33
Consensus LogP	0.41	0.65	0.73	0.78
TPSA (Å^2^)	159.05	159.05	159.05	159.05
Lipinski Violations	1 (heteroatoms > 10)	1 (heteroatoms > 10)	2 (MW > 500; heteroatoms > 10)	2 (MW > 500; heteroatoms > 10)
Bioavailability Score	0.55	0.55	0.17	0.17
GI Absorption	Low	Low	Low	Low
BBB Permeability	No	No	No	No
P-gp Substrate	Yes	Yes	Yes	Yes
CYP3A4 Inhibitor	No	No	No	No
CYP2D6 Inhibitor	No	No	No	No
LD50 (mg/kg)	500	500	500	500
Toxicity Class	4	4	4	4
Hepatotoxicity	Inactive	Inactive	Inactive	Inactive
Mutagenicity	Inactive	Inactive	Inactive	Inactive
Carcinogenicity	Inactive	Inactive	Inactive	Inactive

TPSA: topological polar surface area; GI: gastrointestinal; BBB: blood–brain barrier; P-gp: P-glycoprotein. Lipinski violations were primarily associated with high heteroatom counts and molecular weight. CYP inhibition predictions were obtained from ADMETlab 3.0, whereas LD50 values and toxicity classifications were predicted using ProTox-3.0.

## Data Availability

The data supporting this work are in the article and Supplementary Materials.

## Supplementary Materials

The following supporting information can be downloaded at https://www.mdpi.com/article/10.3390/molecules31132281/s1.
