# Supplementary material for: Novel Sulfonate Derivatives Functionalized with Triazole–Hydrazone Moieties: Synthesis, Characterization, DFT, Targeting Brain Tumors via DNA Damage, Cytotoxicity, Migration Suppression, Antimicrobial Activity, and In Silico Study
Source: Molecules. 2026 Jun 30;31(13):2281. doi: 10.3390/molecules31132281 (PMC13362672; doi:10.3390/molecules31132281)
Supplement: Supplementary file 1 [file molecules-31-02281-s001.zip › molecules-4375622-supplementary.pdf]

Novel Sulfonate Derivatives Functionalized with Triazole–Hydrazone Moieties: Synthesis, Characterization, DFT, Targeting Brain Tumors via DNA Damage, Cytotoxicity, Migration Suppression, Antimicrobial Activity, and In Silico Study

Yasemin Ünver [1,*], Meryem Evecen [2], Fatih Çelik [1], Ali Aydın [3], Halil İbrahim Güler [4], Kadriye İnan Bektaş [4] and Tuğba Usta [1,5]

[1] Department of Chemistry, Faculty of Sciences, Karadeniz Technical University, 61080 Trabzon, Turkey; fatih.celik502@gmail.com (F.Ç.); tugbausta93@gmail.com (T.U.)
[2] Department of Electric and Electronic Engineering, Faculty of Engineering, Amasya University, 05100 Amasya, Turkey; meryem.evecen@amasya.edu.tr
[3] Department of Basic Medical Science, Faculty of Medicine, Bozok University, 66100 Yozgat, Turkey; ali.aydin@yobu.edu.tr
[4] Department of Molecular Biology and Genetics, Faculty of Science, Karadeniz Technical University, 61080 Trabzon, Turkey; hiboguler@gmail.com (H.İ.G.); kadriyensis@gmail.com (K.İ.B.)
[5] Department of Chemistry, Graduate School of Natural and Applied Science, Karadeniz Technical University, 61080 Trabzon, Turkey
* Correspondence: unver.yasemincan@hotmail.com

Table S1

Selected molecular structure parameters of the compounds.

Bond length (Å)	3a	3b	3c	3d
C1-C2	1.49	1.49	1.49	1.49
C2-N3	1.33	1.33	1.33	1.33
C2-N29	1.4	1.4	1.4	1.4
N3-N4	1.42	1.42	1.42	1.42
N4-C5	1.46	1.46	1.46	1.46
N4-C27	1.38	1.38	1.38	1.38
C5-C6	1.55	1.55	1.55	1.55
C6-O7	1.26	1.26	1.26	1.26
C6-N8	1.38	1.38	1.38	1.38
N8-N9	1.39	1.39	1.39	1.39
N9-C10	1.31	1.31	1.31	1.31
C10-C11	1.47	1.47	1.47	1.47
C14-O17	1.39	1.39	1.39	1.39
O17-S18	1.93	1.93	1.93	1.93
S18-O19	1.62	1.62	1.62	1.62
S18-O20	1.62	1.62	1.62	1.62
S18-C21	1.85	1.85	1.85	1.85
C24-X44	1.4	1.81	1.96	2.14
C27-O28	1.26	1.26	1.26	1.26
C27-N29	1.41	1.41	1.41	1.41
N29-N30	1.4	1.4	1.4	1.4
**Bond angles (°)**				
C1-C2-N3	125.3	125.34	125.31	125.3
C1-C2-N29	124.13	124.1	124.12	124.14
N3-C2-N29	110.57	110.56	110.57	110.57
C2-N3-N4	104.89	104.9	104.89	104.89

N3-N4-C5	120.81	120.76	120.83	120.84
N3-N4-C27	112.49	112.47	112.49	112.49
C5-N4-C27	126.61	126.67	126.6	126.59
N4-C5-C6	114.96	115.08	114.96	114.90
C5-C6-O7	120.76	120.72	120.78	120.78
C5-C6-N8	114.59	114.65	114.58	114.57
O7-C6-N8	124.6	124.59	124.6	124.61
C6-N8-N9	129.31	129.31	129.34	129.33
N8-N9-C10	121.77	121.83	121.83	121.77
N9-C10-C11	119.09	119.04	119.02	119.08
C10-C11-C12	122.24	122.21	122.17	122.23
C13-C14-O17	119.49	119.56	119.49	119.48
C14-O17-S18	117.12	117.11	117.07	117.11
O17-S18-O19	108.57	108.76	108.87	108.67
O17-S18-C21	96.07	96.00	96.04	96.02
O19-S18-O20	120.87	120.87	120.83	120.80
O19-S18-C21	109.55	109.53	109.61	109.62
S18-C21-C26	118.12	118.17	118.27	118.25
C23-C24-X44	118.19	118.78	118.1	119.50
N4-C27-O28	128.96	128.98	128.95	128.95
N4-C27-N29	103.04	103.05	103.04	103.03
O28-C27-N29	127.99	127.95	127.99	128.00
C2-N29-C27	109.01	109.02	109.01	109.01
C2-N29-N30	125.49	125.5	125.5	125.49
C27-N29-N30	125.49	125.47	125.48	125.50
**Torsion angles (º)**				
C1-C2-N3-N4	-179.86	-179.86	-179.91	-179.79
N29-C2-N3-N4	0.1	0.13	0.11	0.11
C1-C2-N29-C27	179.84	179.85	179.87	179.79
C1-C2-N29-N30	0.66	0.78	0.86	0.71
N3-C2-N29-C27	-0.12	-0.14	-0.14	-0.17
N3-C2-N29-N30	-179.29	-179.21	-179.15	-179.26
C2-N3-N4-C5	-176.74	-176.69	-176.89	-176.97
C2-N3-N4-C27	-0.05	-0.08	-0.04	-0.02
N3-N4-C5-C6	-111.8	-112.19	-111.69	-111.3
C27-N4-C5-C6	72	71.71	7.93	72.2
N3-N4-C27-O28	178.88	178.85	178.83	178.77
C5-N4-C27-O28	-4.66	-4.77	-4.54	-4.49
C5-N4-C27-N29	176.44	176.37	176.59	176.66
N4-C5-C6-O7	125.21	125.94	125.26	125.17
N4-C5-C6-N8	-57.08	-56.38	-57.03	-57.02
C5-C6-N8-N9	-178.99	-178.14	-178.08	-179.33
O7-C6-N8-N9	-1.38	-1.56	-1.47	-1.62
C6-N8-N9-C10	0.57	1.14	0.72	0.74
N8-N9-C10-C11	179.74	179.8	179.79	179.89
N9-C10-C11-C12	-1.32	-0.45	-1.26	-1.33
C15-C14-O17-S18	88.95	90.51	90.65	89.39
C14-O17-S18-O20	69.84	70.19	65.91	68.22
C14-O17-S18-C21	-176.99	-176.69	179.05	-178.51
O17-S18-C21-C26	88.74	89.40	89.69	89.74
X44-C24-C25-C26	179.91	179.90	179.92	179.92
N4-C27-N29-N30	179.26	179.15	179.12	179.23
O28-C27-N29-N30	0.34	0.28	0.23	0.37

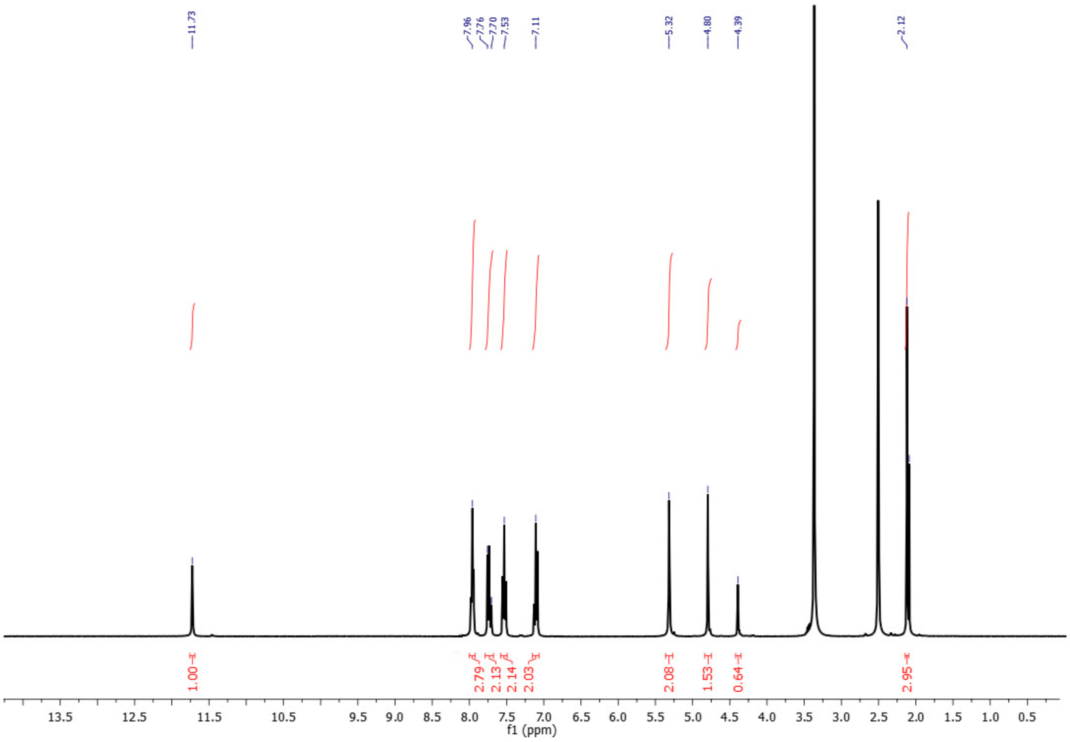

Figure S1. ^{1}H-NMR spectrum of compound 3a.

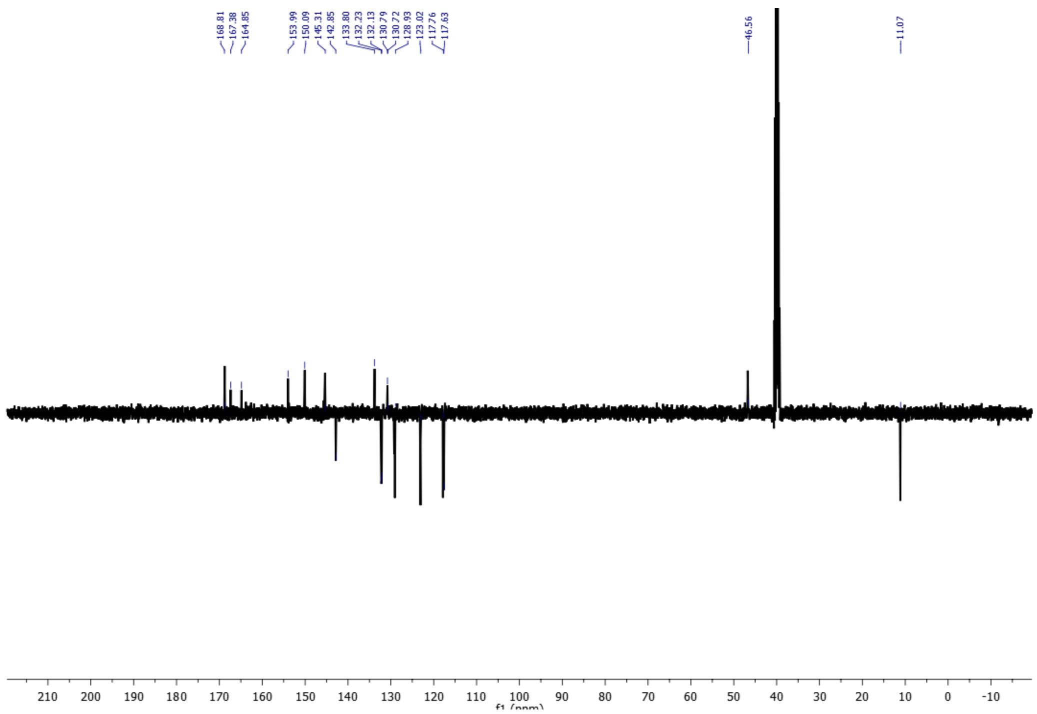

Figure S2. ^{13}C-NMR (APT) spectrum of compound 3a.

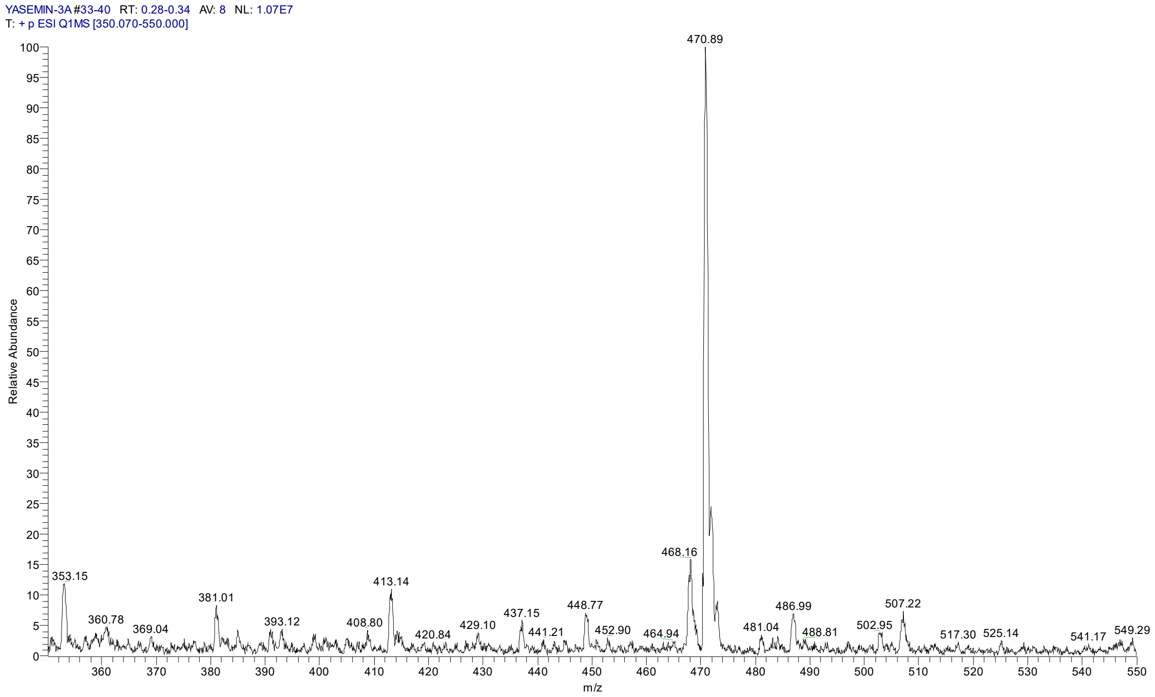

Figure S3. LC-MS/MS spectrum of compound 3a.

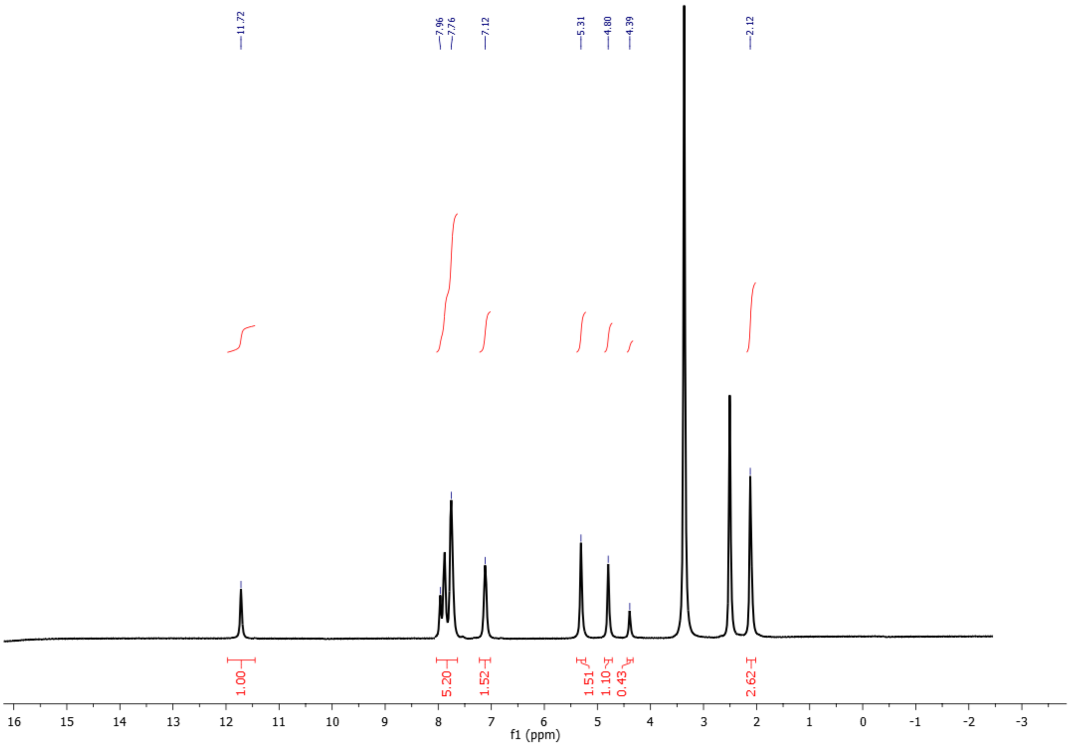

Figure S4. ^{1}H-NMR spectrum of compound 3b.

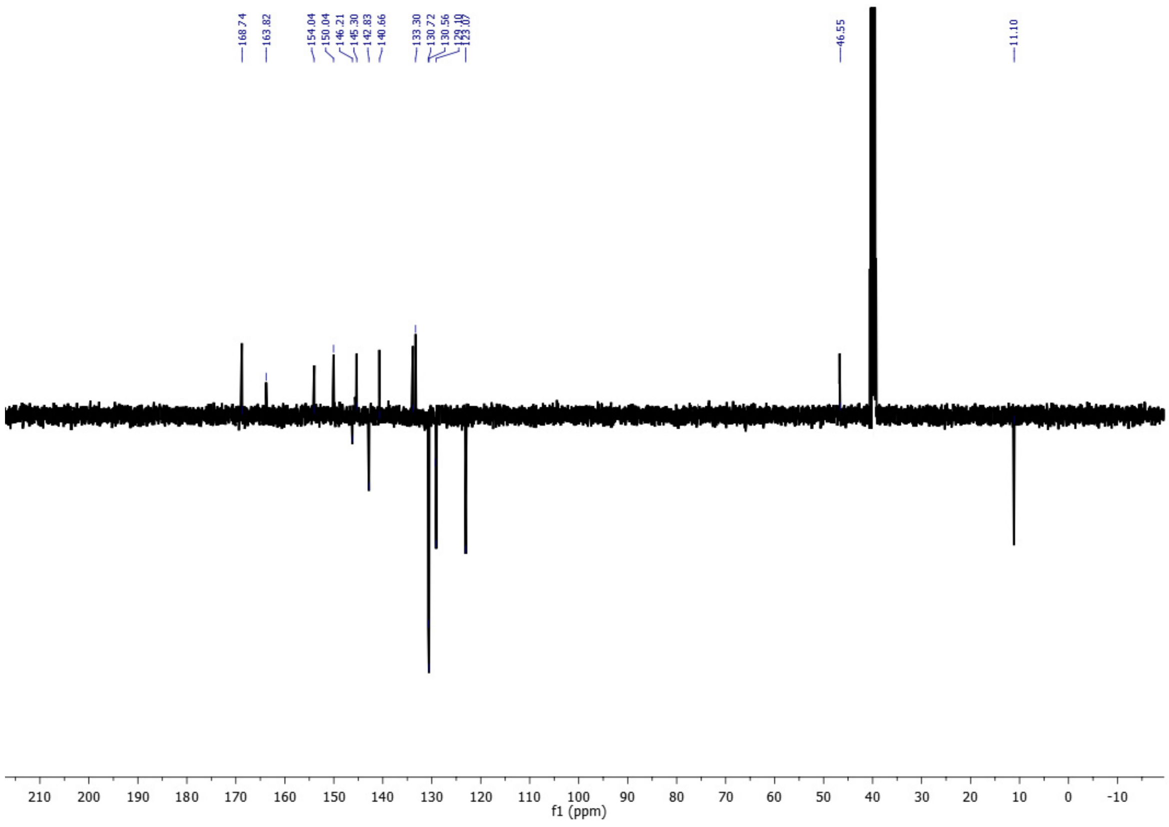

Figure S5. ^{13}C-NMR (APT) spectrum of compound 3b.

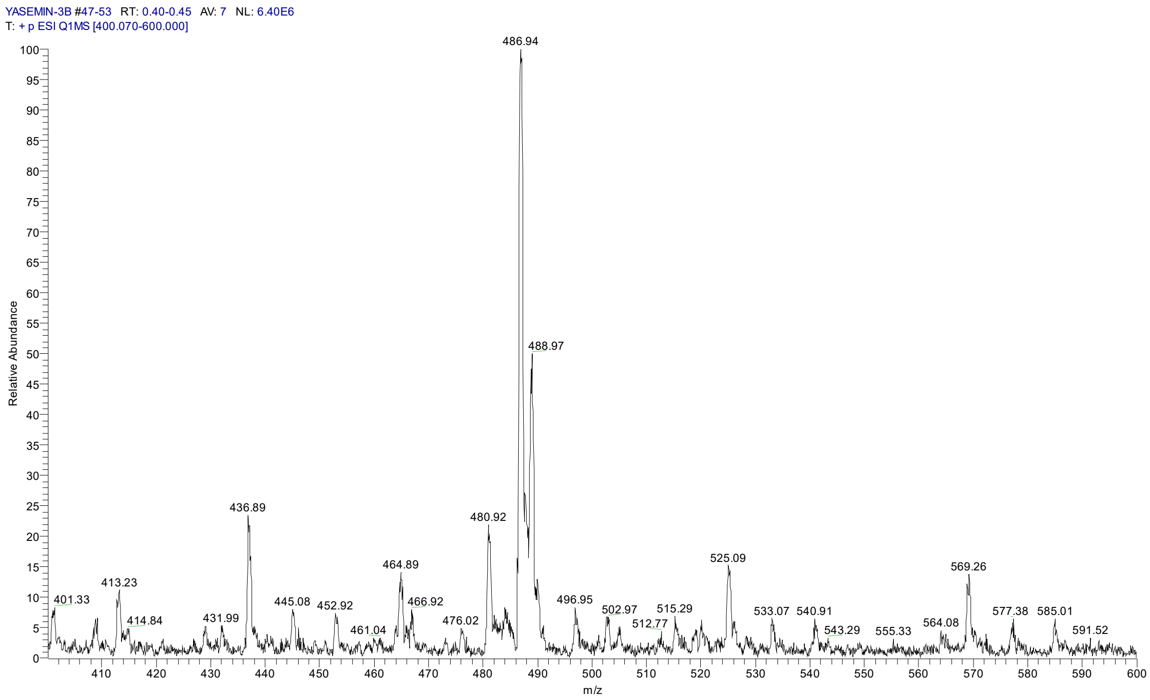

Figure S6. LC-MS/MS spectrum of compound 3b.

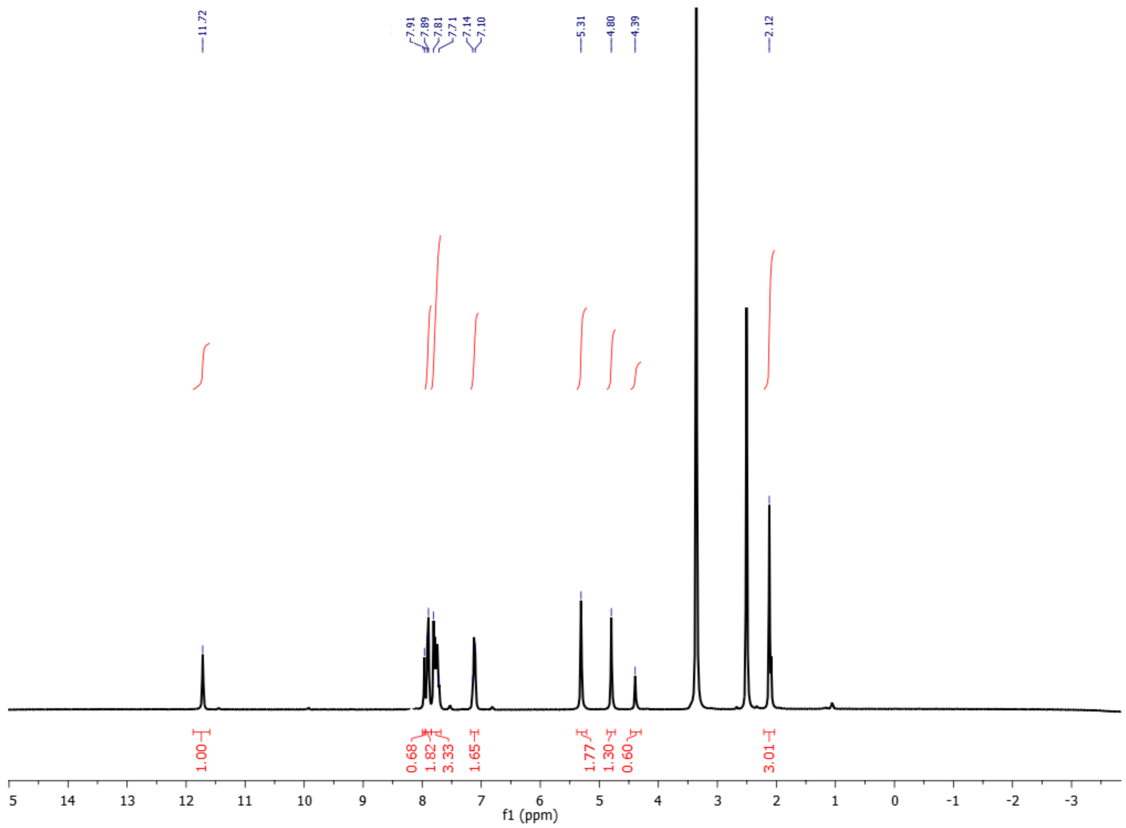

Figure S7. ^{1}H-NMR spectrum of compound 3c.

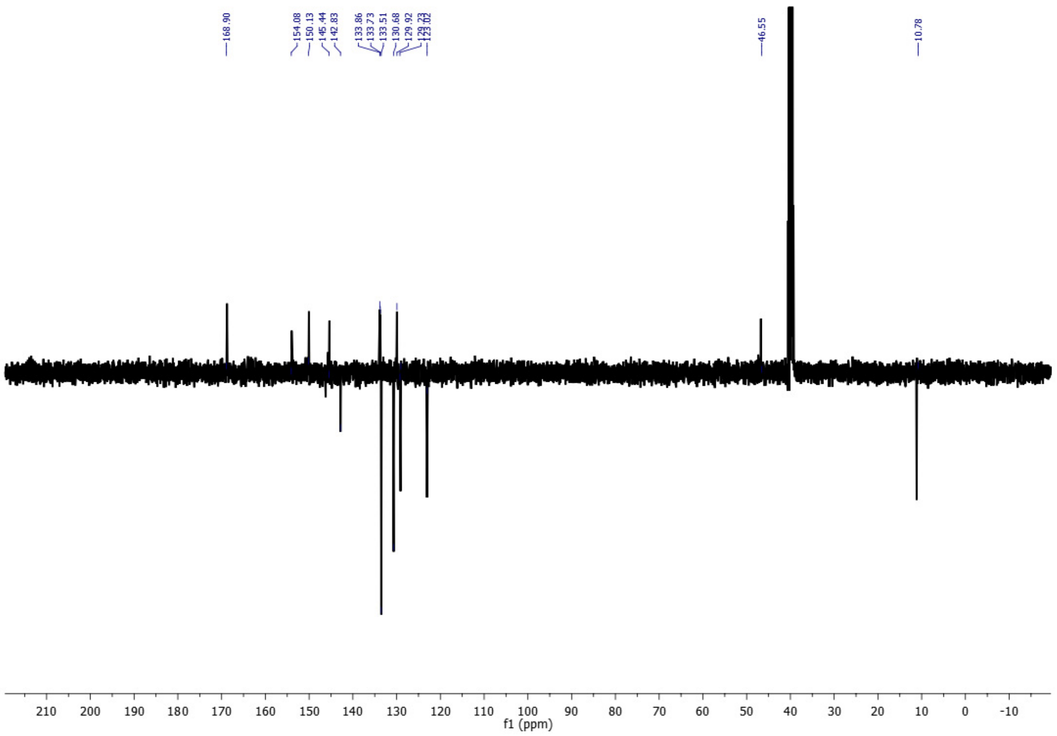

Figure S8. ^{13}C-NMR (APT) spectrum of compound 3c.

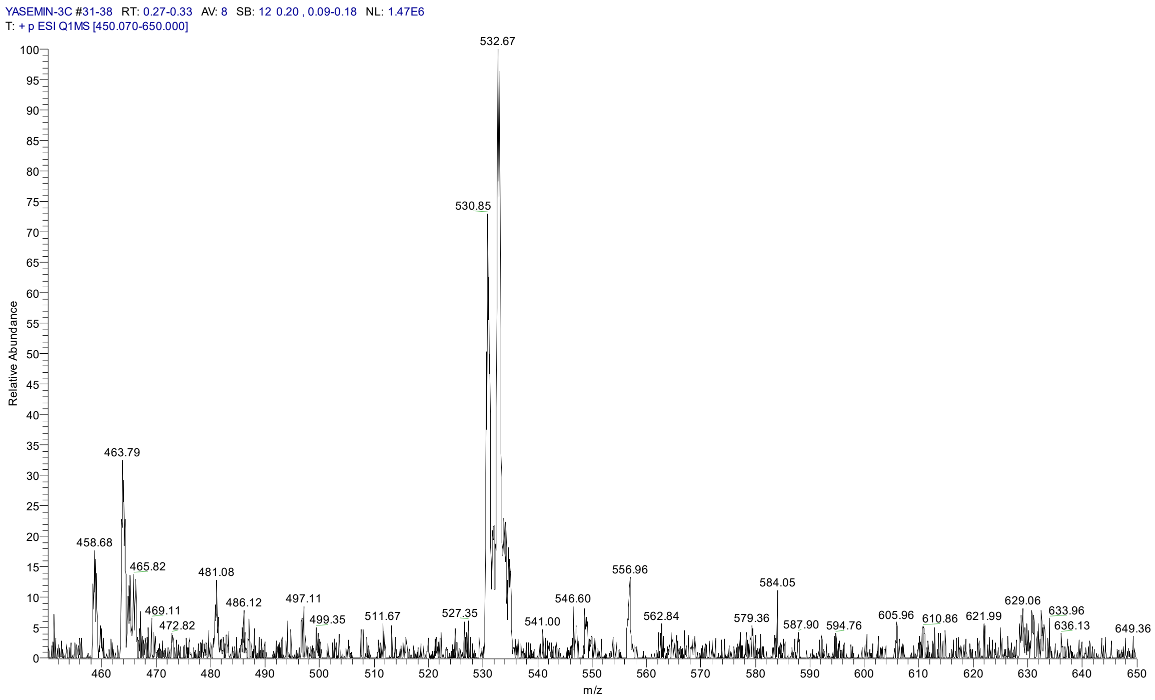

Figure S9. LC-MS/MS spectrum of compound 3c.

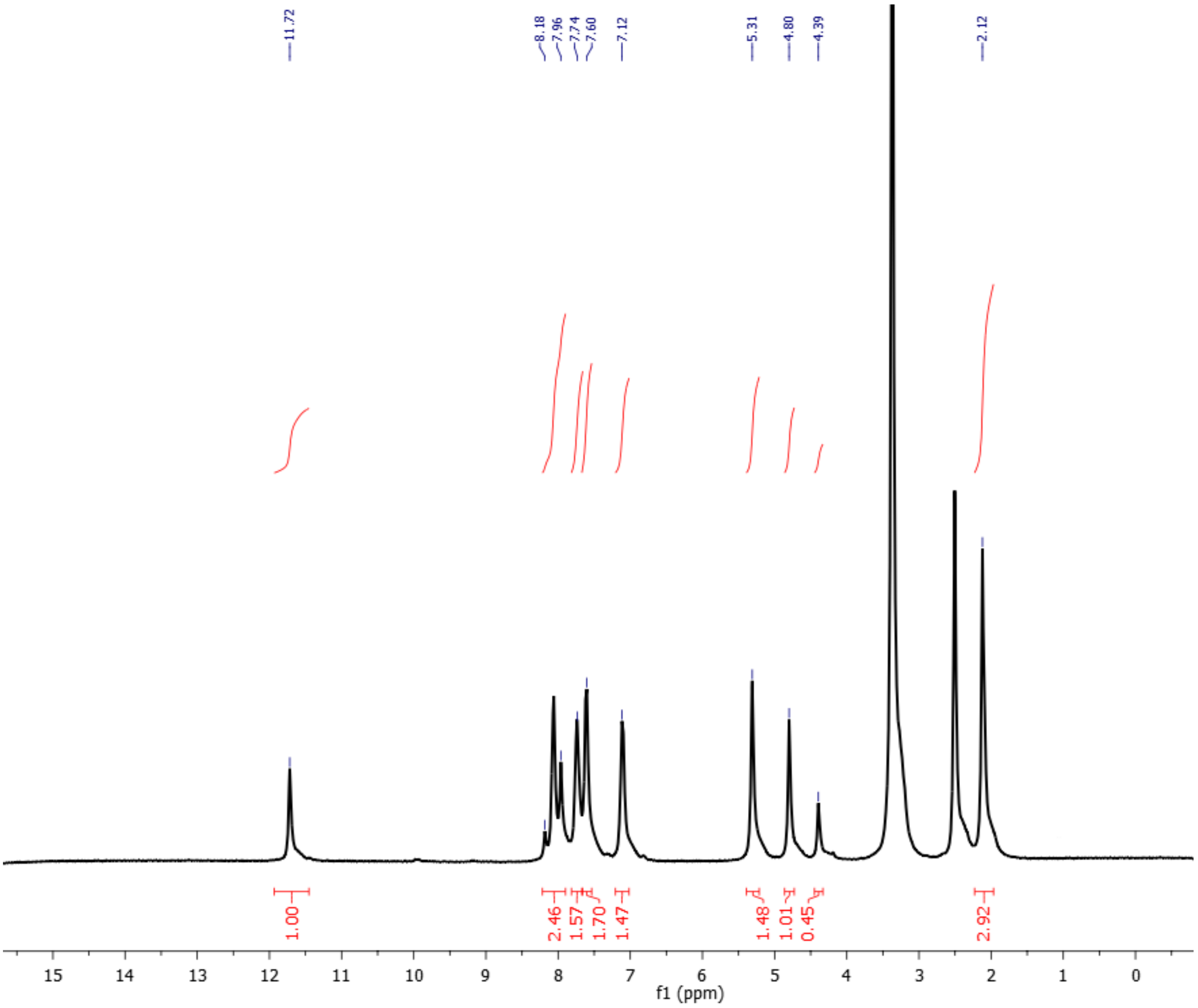

Figure S10. ^{1}H-NMR spectrum of compound 3d.

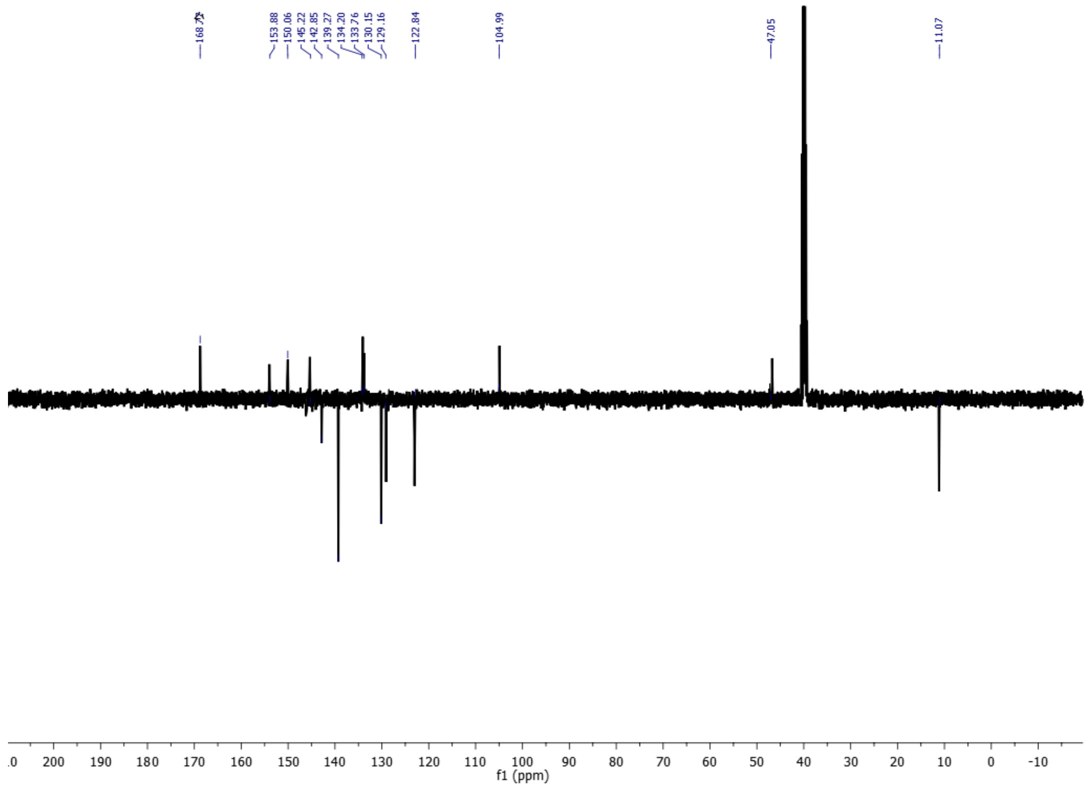

Figure S11. ^{13}C-NMR (APT) spectrum of compound 3d.

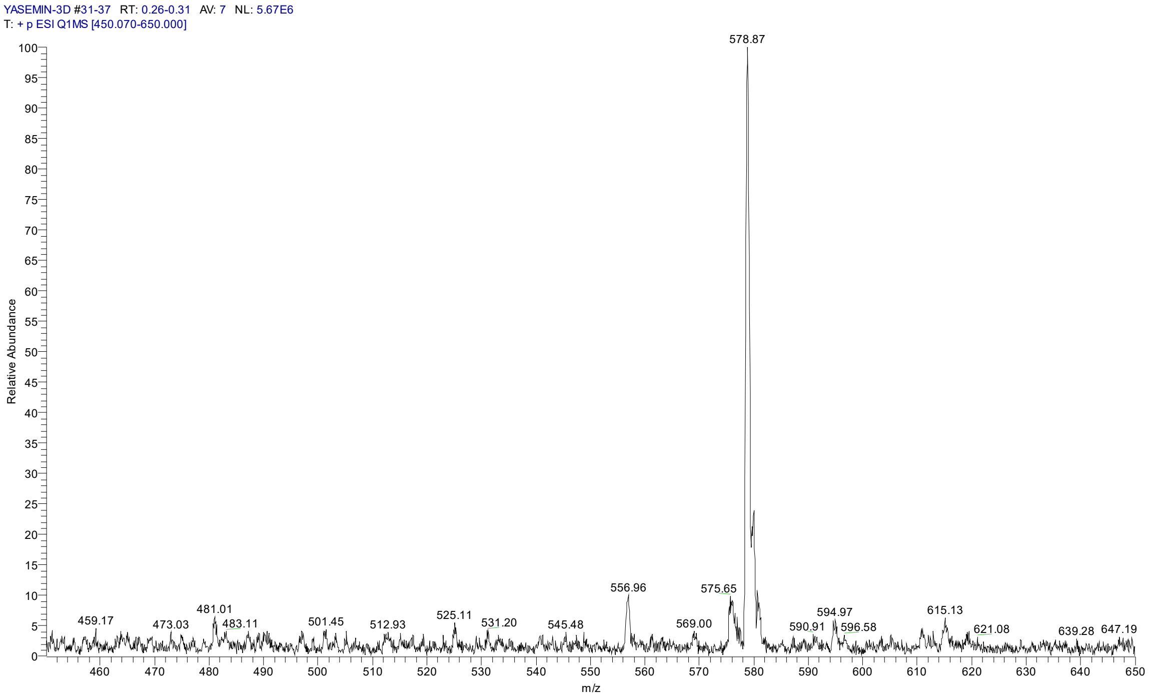

Figure S12. LC-MS/MS spectrum of compound 3d.
